# A Soft Collaborative Robot for Contact‐based Intuitive Human Drag Teaching

**DOI:** 10.1002/advs.202308835

**Published:** 2024-04-22

**Authors:** Shoulu Gong, Wenbo Li, Jiahao Wu, Bohan Feng, Zhiran Yi, Xinyu Guo, Wenming Zhang, Lei Shao

**Affiliations:** ^1^ University of Michigan–Shanghai Jiao Tong University Joint Institute Shanghai Jiao Tong University Shanghai 200240 China; ^2^ School of Mechanical Engineering and State Key Laboratory of Mechanical System and Vibration Shanghai Jiao Tong University Shanghai 200240 China; ^3^ School of Aerospace Engineering and Applied Mechanics Tongji University Shanghai 200092 China

**Keywords:** drag teaching, exceptional repeatability, human collaboration, soft robotics, ultra‐low hysteresis

## Abstract

Soft material‐based robots, known for their safety and compliance, are expected to play an irreplaceable role in human‐robot collaboration. However, this expectation is far from real industrial applications due to their complex programmability and poor motion precision, brought by the super elasticity and large hysteresis of soft materials. Here, a soft collaborative robot (Soft Co‐bot) with intuitive and easy programming by contact‐based drag teaching, and also with exceptional motion repeatability (< 0.30% of body length) and ultra‐low hysteresis (< 2.0%) is reported. Such an unprecedented capability is achieved by a biomimetic antagonistic design within a pneumatic soft robot, in which cables are threaded to servo motors through tension sensors to form a self‐sensing system, thus providing both precise actuation and dragging‐aware collaboration. Hence, the Soft Co‐bots can be first taught by human drag and then precisely repeat various tasks on their own, such as electronics assembling, machine tool installation, etc. The proposed Soft Co‐bots exhibit a high potential for safe and intuitive human‐robot collaboration in unstructured environments, promoting the immediate practical application of soft robots.

## Graphical abstract

The glamorous drag teaching in collaborative robots is for the first time made possible in soft material‐based robots, which are capable of repeating high‐precision industrial tasks after human manual teaching, promoting unrivaled safe and intuitive human‐robot collaboration.

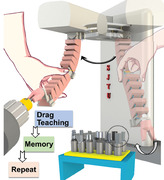

## 1 Introduction

Robots have been replacing humans in hazardous, high‐precision, or tediously repetitive tasks in industry ever since their invention. Recent advancements in collaborative robots, aiming at easier programming and safer operation, have unleashed rigid robots from stringent confinement and segregation from humans. On the other hand, soft robots are particularly developed to address the challenge of safety, as they are intrinsically soft and highly compliant with excellent biocompatibility for both friendly robot‐environment and human‐robot interactions.^[^ ^1^, ^2^, ^3^, ^4^, ^5^ ^]^ However, until now, there are still few studies demonstrating the potential of soft robots for replacing rigid ones in practical industrial applications, leaving them largely for stereotyped tasks in assembly lines such as objects grasping.^[^ ^6^, ^7^, ^8^ ^]^ We envision a scenario in which humans and soft robots sit together at industrial assembly lines to work on collaborative tasks where the human worker teaches the soft robots to perform repetitive tasks by hand guiding. So far to the best of our knowledge, current studies on extending human drag teaching on soft robots or continuum robots are still limited by insufficient accuracy and repeatability and are only for simple motion trajectories.^[^ ^50^, ^51^, ^52^, ^53^ ^]^ We aim to empower soft robots with intuitive and accurate contact‐based human drag teaching capability, broadening its application scenarios.

The first step in operating collaborative robots is to program and record the complete hands‐on drag teaching process, which requires the conversion of prescribed positions into implicit actuating forces or torques. This is an inverse dynamic problem and could only work if the equations of motion are accurately known. Although it works well for traditional rigid robots as their inverse kinematic modeling is straightforward and has real‐time feedback of joint angles and torques, this procedure could not be easily applied to soft robots because their known hyper‐elasticity, large hysteresis and infinite degrees of freedom lead to difficult modeling, perception, and programming. There have been a lot of research efforts in the modeling of soft robots with significant achievements such as in soft parallel robots and soft pneu‐net robots,^[^ ^9^, ^10^ ^]^ but the inverse kinematics of most soft robots are still too complex to model due to the hyper‐elastic properties of soft materials.

The second step in implementing collaboration is to precisely repeat the taught motion in a highly repetitive manner. This is however exactly another weakness of soft robots because precision cannot be guaranteed with soft mechanisms, causing problems such as high overshoot, large hysteresis,^[^ ^11^, ^12^ ^]^ and slow response.^[^ ^13^, ^14^ ^]^ There are indeed lots of research works to address this problem by building closed‐loop control systems to detect and affect their motions in real‐time. Among them, visual servo is the most common and effective way to provide motion and position feedback and is widely used in soft robots of all scales.^[^ ^15^, ^16^, ^17^, ^18^, ^19^, ^20^, ^21^ ^]^ Although they could improve control accuracy and be useful for tele‐operations, visual servo systems do not help the robots to feel how much force a human worker is directly dragging them or do not provide much haptic feedback to the human workers, leading to insufficient human‐robot mutual intuition. Other drawbacks include the difficult handling of imperfect lighting environments, high prices, and limited special working space. Recently, with the development of flexible electronics, various electronic skins (e‐skins) are designed for robotic exteroception ^[^ ^22^, ^23^, ^24^ ^]^ (e.g., tactile, temperature, etc.) and proprioception (e.g., position, angles, etc.),^[^ ^25^, ^26^, ^27^, ^28^ ^]^ leading to advances like closed‐loop wireless motion control,^[^ ^29^, ^30^ ^]^ touchless teaching,^[^ ^31^ ^]^ etc. E‐skins represent tremendous progress toward soft robotic perception and human‐soft robotic interaction, but could only provide a means for contact‐based drag teaching of soft robots after resolving problems such as low‐repetitive responses, delaminated morphing, and non‐discrimination over deformation and tactile pressure, mainly due to the destabilization of soft polymeric materials and the mechanical instability of connecting interfaces.^[^ ^54^, ^55^ ^]^ This results in a repeatability still much worse than those mechanical‐driven rigid robots.

It would be more promising to build from the fundamental level of new structural, control, and perception designs. Hybrid actuation methods are promising for improved soft robotic dynamics by integrating two different actuation schemes for antagonistic stability ^[^ ^32^, ^33^, ^34^ ^]^ and stiffness enhancement,^[^ ^35^, ^36^, ^37^, ^38^, ^39^, ^40^ ^]^ but existing approaches are still far from resolving challenges in human collaboration, programming and repeatability due to the lack of self‐perception and sophisticated control. Here, we propose a soft collaborative robot (Soft Co‐bot) with drag teaching capability, which consists of a soft continuum actuator driven by a pneumatic‐cable antagonistic strategy with on‐demand stiffness enhancement. Most importantly, we add an integrated homemade tension sensing apparatus to constantly monitor the cable tension so that the robot tracks human drag and cooperatively morphs under human guidance by real‐time closed‐loop servomotor control. Such an antagonistic actuation design also directly maps the soft robot's morphing into cable length and servomotor angles, and thus provides a simple modeling scheme for motion programming and later highly precise repetitions. As a result, it also shows ultra‐low hysteresis, oscillation overshoot, and high repeatability compared to typical pneumatic soft robots, cable‐driven continuum robots, or other existing soft robots.

**Figure 1:**
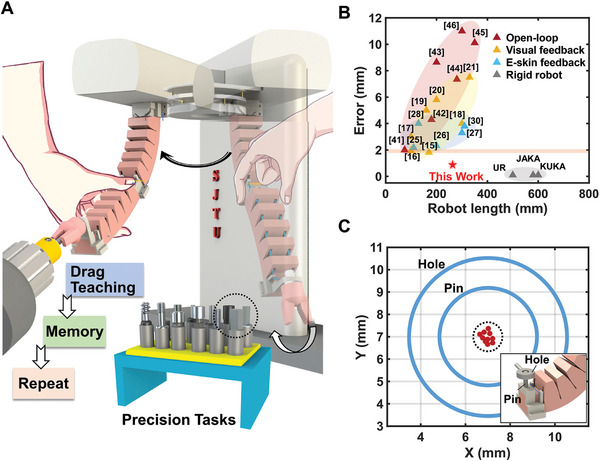
Demonstration of easy programming, high accuracy, and high precision of the contact‐based soft collaborative robot (Soft Co‐bot). A) Soft Co‐bot completes cutter replacement through human drag teaching. B) Comparison of the proposed drag teaching method's positioning error (robot accuracy) with previously developed soft robots’ control strategies, including those with open‐loop control strategies (41–46), visual‐based feedback control strategies (15–21), and electronic‐skin based feedback control strategies (25–28 and 30). C) Demonstration of the high precision for this proposed Soft Co‐bot in performing a repetitive task (pin latching).

As shown in **Figure** **1**A, the Soft Co‐bot could repetitively complete machine tool retrieval and replacement tasks after human drag teaching without any visual systems or e‐skins. This is distinctly different from most other works in soft robots that emphasize remote or wireless feedback, but instead, simply rely on a much more intuitive contact‐based collaboration. Notably, this Soft Co‐bot exhibits extremely high absolute positioning accuracy (less than 1 mm out of a 230 mm body length) compared to current soft robots with either open–loop control ^[^ ^41^, ^42^, ^43^, ^44^, ^45^, ^46^ ^]^ or closed‐loop control ^[^ ^15^, ^16^, ^17^, ^18^, ^19^, ^20^, ^21^, ^22^, ^23^, ^24^, ^25^, ^26^, ^27^, ^28^, ^29^, ^30^, ^31^ ^]^ (Figure 1B), and is approaching the accuracy of advanced commercial rigid robots. We then demonstrate that this Soft Co‐bot can memorize the motion trajectory after drag teaching and repeat tasks with high precision on its own (Figure 1C, maximum absolute positioning error between the end marker points of the Soft Co‐bot less than 1 mm for repeating 15 times). In the following, we detail the design of the antagonistic actuation, the human‐robot drag teaching system, and then the characterization of the easy drag programming followed by excellent actuation performance. Finally, we show various repetitive industrial tasks after a single human teaching step, such as bolt positioning, electronics assembling, and cutter tool replacement, which stand from all previous soft robots in human‐robot collaboration.

## 2 Results

### 2.1 Design of Antagonistic Actuation

**Figure 2:**
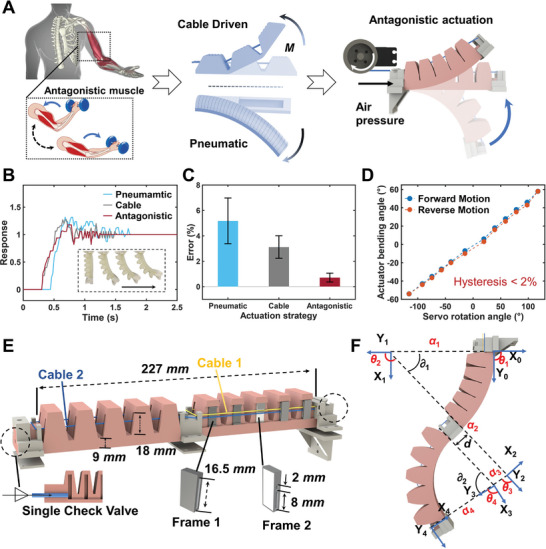
Design, structure, and actuation performance of the antagonistic soft continuum actuator. A) Antagonistic actuation (combination of pneumatic and cable‐driven) inspired by human‐antagonistic muscles. B) Measured motion response (normalized results) as a function of actuation time under pneumatic actuation, cable‐driven actuation, and antagonistic actuation. C) Measured average motion errors of repeatability test for 50 cycles under pneumatic actuation, cable‐driven actuation, and antagonistic actuation. D) Measured bending angles of the antagonistic actuator as a function of servo rotation angles during one motion cycle, showing an ultra‐low hysteresis between the forward and reverse bending processes. E) Structure and geometric parameters of the soft continuum actuator. F) Modeling for the relationship between the bending angles and end positions of the soft continuum actuator.

Inspired by antagonistic contraction and stiffness tuning of muscles in human arms, we design a soft actuator combining pneumatic bending and cable pulling with opposed drive effects to achieve an antagonistic pair (**Figure** **2**A) and straightforward modeling. We define the pneumatic bending as positive motion while the cable pulled bending as negative motion for later discussions. While the driving cable guarantees precise kinematics by its length, the output force and transient dynamics of the actuator require stiffening via an adjustment on the inflation pressure. The essence of the stiffness enhancement of the actuator lies in the extra pneumatic work which increases the potential energy and thus the total energy of the actuator, which can be expressed as:

(1) $$U_{total}=U_{bending}+U_{strain}+\Delta PV$$

In which, *U*_bending_ and *U*_strain_ represent the bending energy and the stretching energy in the soft bending actuator, and Δ*PV* is the increased pneumatic work. After comprehensive consideration, we chose the pre‐inflation pressure to be 80 kPa (specific reason described in Supporting Text 4). The enhanced stiffness greatly improves the transient dynamics of the actuator, resulting in a better dynamic performance compared to other soft actuators (e.g., pneumatic network, sole cable‐driven, shape‐memory, etc.).

Figure 2B shows a typical measured step response by a thin film sensor (sample rate 50 Hz) of the actuator in antagonistic actuation mode (in red), a pure pneumatic actuation mode (in blue), and a pure cable‐driven actuation mode (in grey). Notably, the antagonistic actuation mode shows less overshoot (< 8%), faster rise time (< 0.2 s), and shorter settling time (< 0.45 s). We also record the position errors of the end marker point on the actuator to characterize the motion repeatability by setting the first cycle as the reference point. Figure 2C shows the measured average motion errors for 50 cycles, demonstrating a significantly improved motion repeatability with an average motion error of less than 2% for the antagonistic actuation mode. Figure 2D maps the motor angle into the bending angle of the actuator, showing an ultra‐low hysteresis (< 2.0%) between the forward and reverse bending processes, which is a significant improvement compared to other soft materials and actuators (More in Table S3, Supporting Information).

### 2.2 Design and Open‐Loop Operation of the Soft Co‐bot

The robot body of the Soft Co‐bot is a two‐section soft continuum actuator, with the assembly method detailed in Figure 2E (Fabrication of the proposed Soft Co‐bot in Experimental section). Each actuator has 5 net airbags, and each of them contains an air chamber in the center and two separate narrow slots at the two sides for installing frames to guide the cables. Two different frames are designed, where Frame 1 has only one long and narrow slit opening (in gray) and Frame 2 has a 2‐mm small opening and a short narrow slit opening (in white). The small opening in Frame 2 prevents contact between the cable and the actuator body, ensuring both accurate kinematic modeling and low motional hysteresis (More in Supporting Text 6). For each section of the soft continuum actuator, different frames are inserted into the slots at the two sides in the same particular order to thread the cables, where Frames 2 are placed in the middle net airbags and Frames 1 are placed in other net airbags. Two cables are threaded through the openings in the frames, as a yellow cable (Cable 1) is utilized to actuate the upper half section (one end fixed to the middle connection part and the other wound to a servo motor), and a blue cable (Cable 2) is utilized to actuate the lower half section (one end fixed to the end tip and the other end wound to another servo motor). After assembling, we first inflate the actuators through a single check valve to increase its initial pressure, and then control the robot motion by actuating the two servos by reeling in or reeling out the cables (Figure S11, Supporting Information).

To understand the programming and control of the Soft Co‐bot, we perform kinematic modeling of the continuum actuator body based on a piecewise constant curvature assumption and implement it for more complex tasks. In Figure 2F, we set the bending angles of the two sections to be *∂*_1_ and *∂*_2_, *X*_i_ *OY*_i_ the coordinate on different joints (*X*_0_ *OY*_0_ the base coordinate), *α*_i_ the distance between joints, *θ*_i_ the rotation angles between different coordinates. Therefore, the transition matrix from coordinate i to i+1 can be expressed as:

(2) $$T_{i+1}^{i}\left(\theta_{i+1}\right)=\left[\begin{array}{ccc} cos\theta_{i+1} & -sin\theta_{i+1} & a_{i+1}cos\theta_{i+1}\\ sin\theta_{i+1} & cos\theta_{i+1} & a_{i+1}sin\theta_{i+1}\\ 0 & 0 & 1 \end{array}\right]$$

The position of the endpoint (x_4_, y_4_) in the base coordinate *X*_0_ *OY*_0_ can be calculated based on the position (x_4_, y_4_) on its own coordinate *X*_4_ *OY*_4_ and the total transition matrix (More in Supporting Text 7):

(3) $$\left[\begin{array}{c} x_{0}\\ y_{0}\\ 1 \end{array}\right]=\prod_{i=0}^{3}T_{i+1}^{i}\;\left(\theta_{i+1}\right)\left[\begin{array}{c} x_{4}\\ y_{4}\\ 1 \end{array}\right]$$

We note that the enhanced stiffness via antagonistic actuation can greatly improve the motion independence between the two sections of the soft continuum robot (Movie S1, Supporting Information), thus improving the position control accuracy (Figure S14, Supporting Information, and more in Supporting Text 8). In open‐loop point‐to‐point tracking experiments, the Soft Co‐bot shows a high open‐loop accuracy with an average error ≈1.5% (offset distance / body length) (Figure S16, Supporting Information).

**Figure 3:**
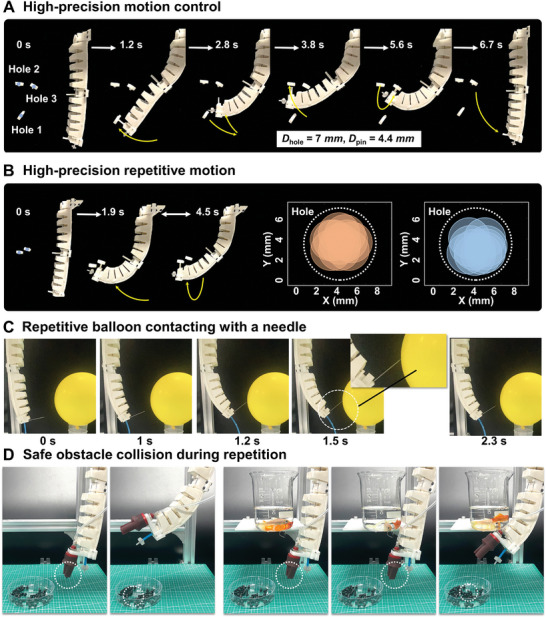
High‐precision repetitions in various working spaces based on open‐loop control. A) Continuous pin latching for three adjacent holes in narrow space within 7 s. B) Highly repeatable pin latching between two adjacent holes with repeated positioning errors of 1 and 0.9 mm, respectively. C) Soft Co‐bot repeatedly contacts a balloon with a sharp needle. D) Soft Co‐bot interacts with a fragile beaker, showing friendly environment‐machine interaction.

We then show the high‐precision repetitive motion of this Soft Co‐bot by an open‐loop operation for a series of demonstrations. **Figure** **3**A shows a continuous and rapid pin latching operation for three adjacent holes (diameter of hole and pin are 7 and 4.4 mm, respectively, Movie S2, Supporting Information). Figure 3B shows that the pin is actuated to move between two holes back and forth rapidly in a highly accurate and stable manner, resulting in average re‐positioning errors of two holes to be 1 and 0.9 mm, respectively (15 times). Figure 3C shows a highly repetitive positioning of a needle installed as the end effector of the Soft Co‐bot, where the needle repeatedly touches a balloon but does not pop it because of precise motion control (Movie S3, Supporting Information). This repetitive positioning error proves its capability towards complex and highly reliable motions.

To further demonstrate the advantages and intrinsic adaption of the Soft Co‐bot in variable unstructured environments compared to the rigid robots, we show that the Soft Co‐bot performs a pick and place task repeatedly in different environments (Figure 3D). Here we place an obstacle (a beaker with two goldfish) on the pre‐programmed motion trajectories of the robot. It shows that the Soft Co‐bot can still repeat the pick and place task even with the obstacle in its pathway. Even though the collision between the Soft Co‐bot and the beaker caused the goldfishes to swim in fear, both the beaker and the robot were intact (while a rigid collaborative robot smashes the beaker, so it needs to be confined in a certain zone for safe operations), showing the friendly environment‐robot interaction of the Soft Co‐bot, as well as more potential application scenarios than rigid robots (Movie S4, Supporting Information).

### 2.3 Drag Teaching System

**Figure 4:**
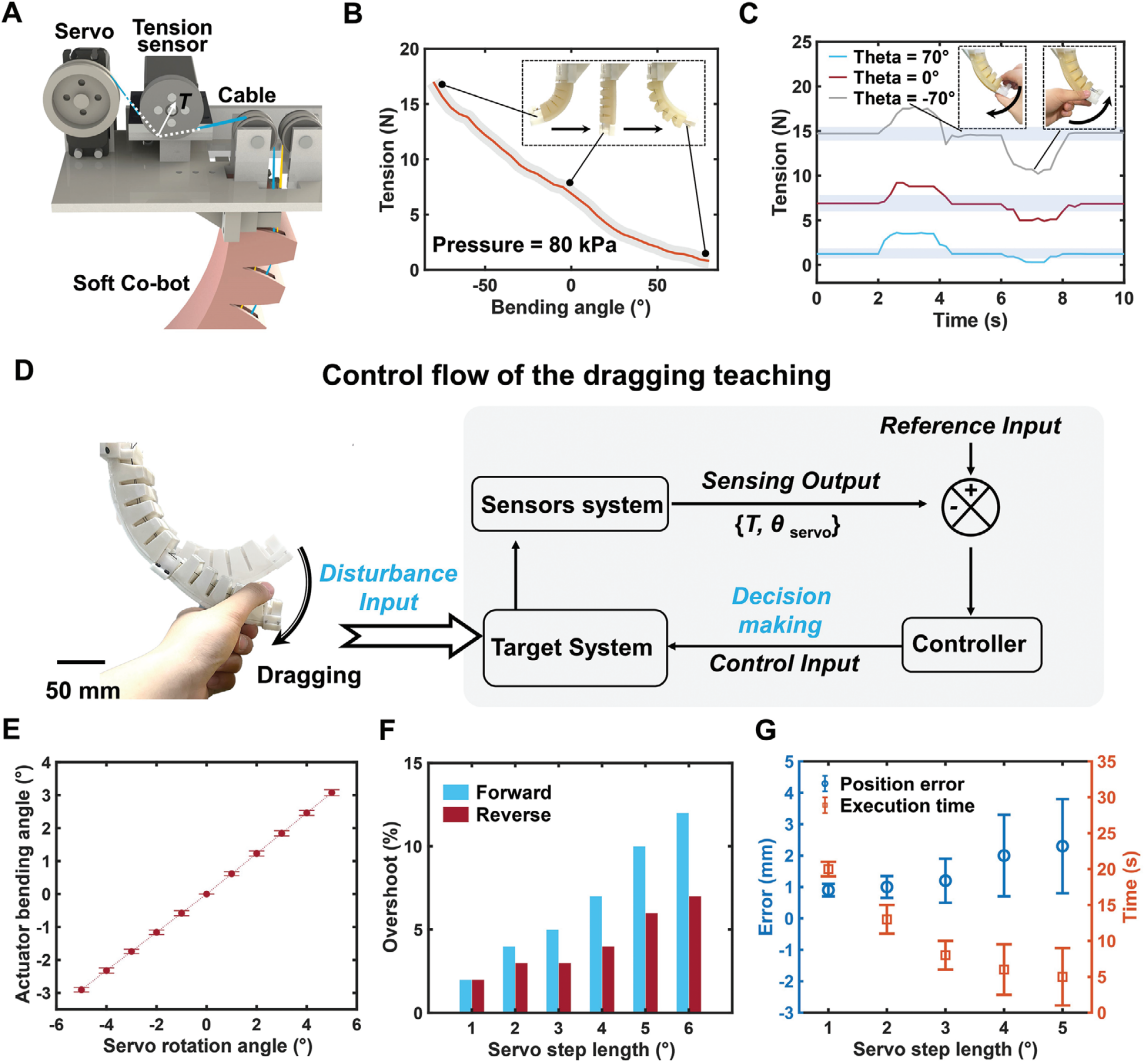
Collaborative control strategy for the Soft Co‐bot. A) Schematic diagram of cable tension measurement. B) Measured cable tension at different bending angles of a single antagonistic actuator. C) Measured cable tension variation of the actuator at different bending angles (−70 degrees, 0 degrees, and 70 degrees) under external drag. D) The closed‐loop control framework for collaborative operation. E) Measured motional resolution as a function of servo rotation angles (servo step length). F) Measured response overshoot of the actuator at different servo step lengths with a sample rate of 10 Hz. G) Measured position error and execution time when the user performs the same drag teaching for the Soft Co‐bot under different servo step lengths.

Our unique Soft Co‐bot design also enables direct contact‐based drag teaching by adding closed‐loop control to the cables. The basis for implementing drag teaching is a tension measurement system, constantly monitoring and compensating the tension in cables (**Figure** **4**A), which contains tension sensors, D/A modules, and a controller processor (Figure S18, Supporting Information). Two tension‐sensing apparatuses are built to detect the tension variation in the two cables. We first measure the tension variation of a single actuator for 20 motion cycles (from −80° to 80° with a servo rotation speed 0.1 rad/s). As shown in Figure 4B, there is a high consistency in the relationship between cable tension variations and actuator bending angles (gray shade indicating the error bar), which is due to the high motion repeatability and low hysteresis by the antagonistic actuation method. We notice that the corresponding cable tension of the actuator at different bending angles is quite stable under no external disturbances, and we further show whether the actuator can identify the directions of external disturbance through the feedback of tension variations. Figure 4C shows measured cable tension variations of the same single actuator at 3 representative bending angles (−70°, 0°, and 70°) under different human hands‐on drag which is treated as external disturbances. We note that the dragging force is small so the actuator shape could be assumed as no variation. Experimental results show that the cable tension clearly increases when the drag direction is along the positive bending direction of the actuator (defined as forward drag), and similarly, the cable tension will decrease when the drag direction is along the negative bending direction of the actuator (defined as reverse drag). When the dragging force is withdrawn, the cable tension will rapidly revert to the original value (Figure S19, Supporting Information).

Combining the characterized relationship between actuator angle, cable tension, and human dragging force as preparation, we design a control algorithm based on the tension sensor signals and servo motor readouts to detect the drag direction in real, and then actuate the robot along the drag direction accordingly until the drag is withdrawn (Figure 4D). Such a scheme achieves reliable motion recording and programming of the robot by simple collaborative drag (More in Supporting Text 9), which empowers the soft continuum robots into a true collaborative robot programmable by drag teaching. During the drag teaching process, the motion resolution of the Soft Co‐bot is determined by its bending step length (minimal bending angle of each section), which is directly related to the robotic positioning accuracy. As shown in Figure 4E, with a single control pulse, the smaller the servo rotation angle is, the smaller the actuator bending angle variation will be, which represents a higher motion resolution for the Soft Co‐bot. But simultaneously, the dynamic speed of the Soft Co‐bot will be reduced proportionally. Therefore, the selection of step length should consider both accuracy and efficiency. After considering the processing capability of both the servo motors and the control board, the sampling rate of the collaborative robot for dragging judgment is set at 10 Hz to ensure accurate drag identification and reliable robotic motion (More in Supporting Text 9). Second, we need to choose the servo step size, which will lead to a larger peak overshoot for a greater step length, due to the rotational inertia of soft materials. This is captured in Figure 4F, as it shows the measured overshoot under different servo step lengths for both forward bending and reverse bending, by actuating the upper half section of the Soft Co‐bot. Third, we measure the steady‐state positioning error of the Soft Co‐bot under a step input at different servo step lengths from 1 to 5 degrees. As shown in Figure 4G, although a large servo step length brings higher motion efficiency, the associated error is greater, and correspondingly, the small servo step length brings high positioning accuracy but low task efficiency. After considering all these trade‐offs, we choose the servo motion step length to be 3 degrees to ensure both efficiency and accuracy.

**Figure 5:**
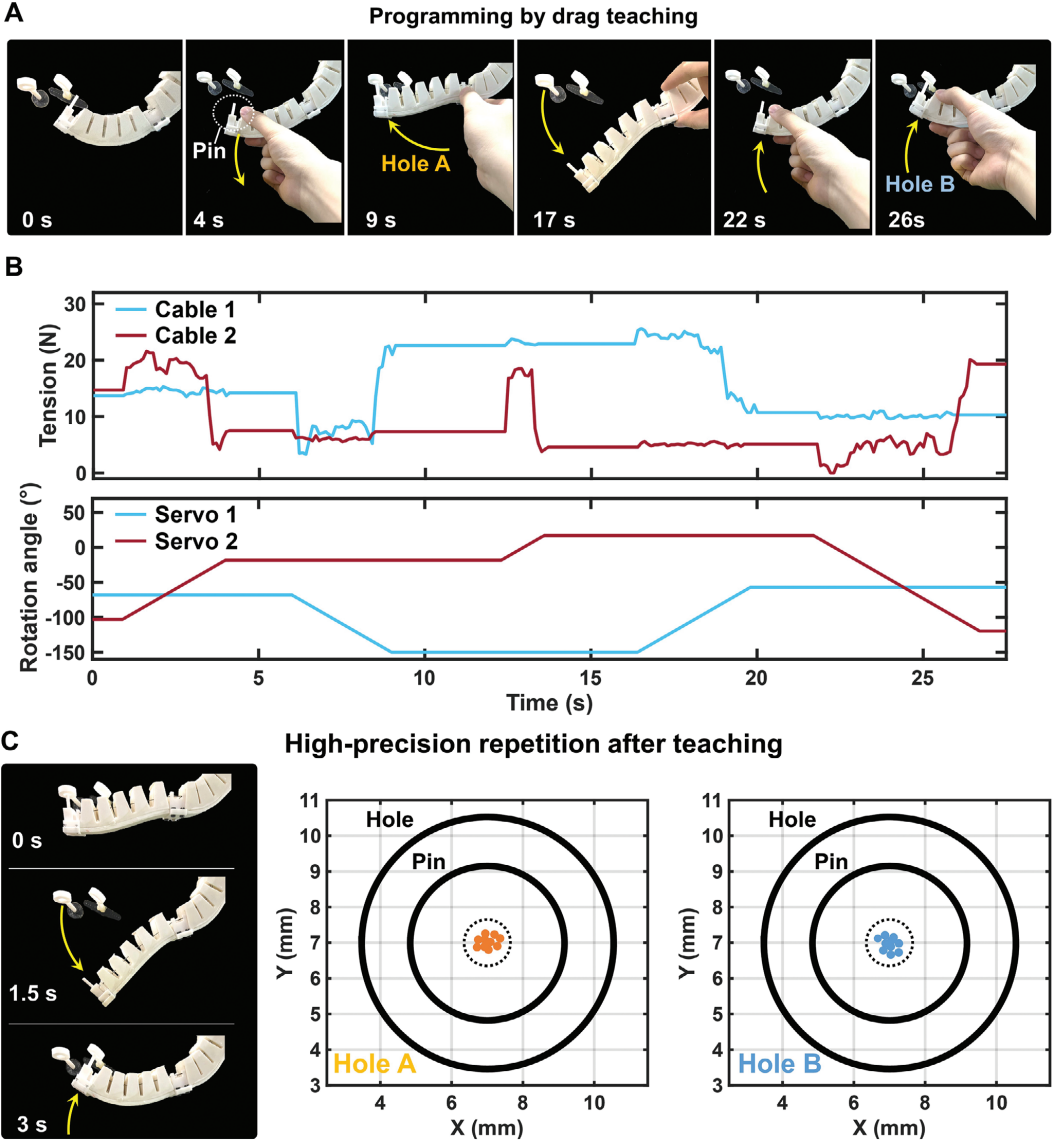
Collaborative and repeated operation with the Soft Co‐bot for pin‐latching tasks. A) Soft Co‐bot performs rapid pin latching operation for different holes based on drag teaching. B) Monitored tension sensors and servo rotation angles of the Soft Co‐bot during the complete drag teaching process. C) Highly repetitive pin latching operation after drag teaching. The average positioning errors of the Soft Co‐bot for Hole A and Hole B for 10 trials are 0.7 and 0.8 mm, respectively.

Based on the proposed control algorithm and optimized parameters, we now show the complete procedure on how to drag and teach the Soft Co‐bot for various different industrial tasks (Movie S5, Supporting Information). **Figure** **5**A shows the programming process of the Soft Co‐bot to precisely latch through two holes by drag teaching. The whole teaching process contains two stages. In the first stage (from 0 to 9 s), we first forward drag the lower half section of the Soft Co‐bot, and at the same time, the Soft Co‐bot detects the external drag and accordingly actuates its lower half section to track the dragging. When the Soft Co‐bot moves to the suitable position, we withdraw the dragging force. We then reverse‐drag the upper half section of the Soft Co‐bot to implement the pin‐latching operation through Hole A. Then, in the second stage (from 10 to 26 s), we drag the Soft Co‐bot to move the carried pin out of Hole A and move to the suitable position for the subsequent pin latching operation for Hole B by repeatedly dragging the two sections of the Soft Co‐bot. After that, we reverse dragged the Soft Co‐bot to control the carried pin to approach Hole B and finalize the pin latching movement in the end. Figure 5B shows the monitored tension variations of two cables and the rotation angles of two servos during the human drag process. We note that the whole teaching process requires no external devices or sensors, but relies only on human hand manipulation and visual feedback.

We note that the antagonistic actuation greatly improves the stability of the Soft Co‐bot during interaction with a user (Figure S17, Supporting Information). When we drag on one section of the Soft Co‐bot, the shape or position of the other section will not be affected. After the whole drag teaching process, the potentiometers in servos could memorize and thus program the time sequence of the rotation angles, and thus the Soft Co‐bot can repeat the taught tasks (Figure 5C). Finally, we demonstrate that the taught motion could be repeated with high speed and high precision, as the average positioning errors for the two targets are ≈0.7 and 0.8 mm in ten trials (0.30% and 0.35% in terms of body length), respectively. These results show the potential that the Soft Co‐bot with drag teaching could possess the same fast programming capability and excellent repeatability as a rigid collaborative robot.

### 2.4 Soft Co‐bot for Complex Industrial Tasks

For broader application scenarios, we add a top turntable to mount the Soft Co‐bot to broaden its 2D planar motion to 3D space and have also designed the corresponding control algorithm to realize the turntable rotation collaboration while dragging the Soft Co‐bot (Figures S21 and S22, and more in Supporting Text 10). Then, we further demonstrate how a user can teach the Soft Co‐bot to complete even more complex industrial assembling tasks by simple hands‐on dragging.

**Figure 6:**
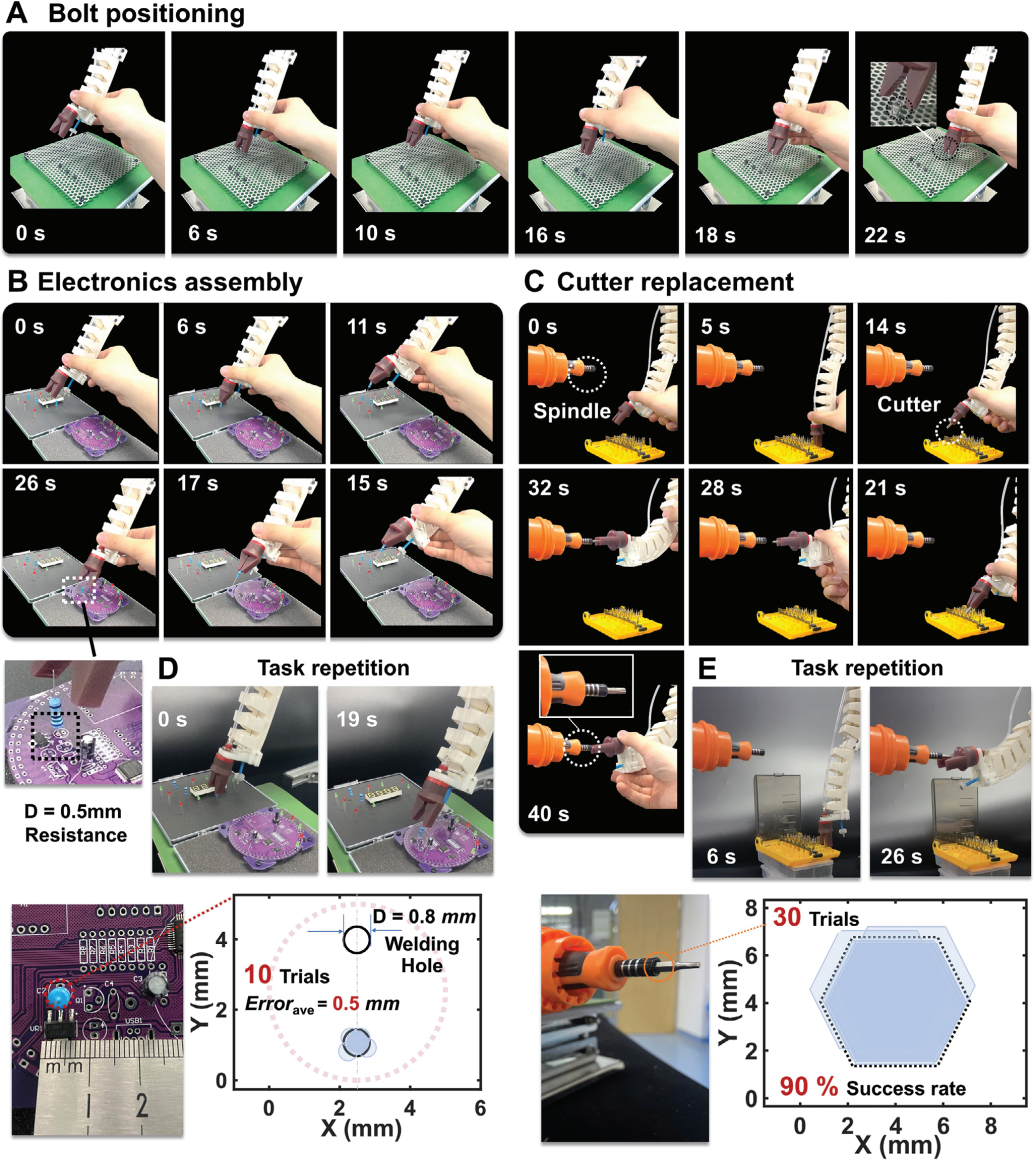
Soft Co‐bot completing complex assembly tasks by contact‐based drag teaching. A) Soft Co‐bot for repetitive bolt assembly based on drag teaching. B) Soft Co‐bot for repetitive electronic component assembly based on drag teaching. C) Soft Co‐bot for repetitive cutter replacement based on drag teaching. D) Highly precise repeated operation for electronics assembling. The average positioning error of 10 trials after drag teaching is ≈0.5 mm. E) Soft Co‐bot for highly precise repeated cutter replacement after drag teaching, and the successful rate of 30 trials is 90%.

The human‐robot drag teaching process can be divided into three steps. In step 1), the user drags the Soft Co‐bot to grasp a part; In step 2), the user drags the Soft Co‐bot to explore a suitable motion path toward the target; In step 3), the user drags the Soft Co‐bot to place the part in the target position. According to such a spirit, we have constructed various complex application scenarios to emphasize the powerful performance of the Soft Co‐bot. The first scenario is “Bolt positioning”, **Figure** **6**a shows the interactive teaching process between the user and the Soft Co‐bot. this process can be divided into 3 stages in time sequence: 1) we drag the Soft Co‐bot to gradually approach the bolt, and when the Soft Co‐bot is dragged to a proper position, the end gripper is manually controlled to pull out the bolt (from 0 to 6 s); 2) we then drag the Soft Co‐bot so the turntable moves and stops at a proper position to transfer the bolt to the target hole (from 6 to 16 s); and 3) we finally drag the Soft Co‐bot to move the bolt to the target hole and when the bolt is aligned with the target hole, the robotic gripper release the bolt (from 16 to 22 s). After teaching, the Soft Co‐bot and end gripper can repeat the task independently (Movie S6, Supporting Information). The whole control process is based on the users’ visual feedback and hand manipulation, so the programming of such an intuitive human‐robot interactive teaching approach is quite fast and accurate even facing complex tasks in an unstructured environment.

As the tasks become more difficult (e.g., more elaborate targets, more complex environments, etc.), collaborative operations become more tedious and time‐consuming, but it is still accurate and reliable. We then demonstrate that the Soft Co‐bot can perform finer tasks, such as small electronics assembly and cutter replacement, which are almost comparable to advanced rigid co‐bots. The first attempted task is electronics assembly, and we anticipate the Soft Co‐bot to be deployed for small‐scale electronic workshops. Here, taking the assembly of a pin resistor as an example, in which the resistor pin and target welding hole are 0.5 and 0.8 mm in diameter, respectively. The whole collaboration process can also be divided into 3 stages: 1) Grab the resistor (from 0 to 6 s); 2) Transfer the resistor (from 6 to 15 s); and 3) Insert the resistor into the target welding hole (from 15 to 26 s). For such fine tasks, the last two stages take more time, and the user needs to adjust the Soft Co‐bot repeatedly to reach the target position. After teaching, we also test the repeatability of the pre‐programmed task and record the resistor pin locations at the end of the assembly in ten consecutive experiments (Movie S7, Supporting Information), and the success rate of resistor grasping tasks is 80% with a commercial gripper. The measured average offset between the resistor pin and the target welding hole is only ≈0.5 mm for 10 trials (Figure 6D), showing that the Soft Co‐bot is capable of executing fine tasks reliably and repeatedly.

The second attempted task is cutter replacement, which is a dangerous and delicate task that is not expected to involve human operation. It is also hard for rigid robots, as a small misalignment could result in a mechanical hard contact and a great contact force, leading to damages to cutters or spindles, but this is not a concern for soft robots due to their high compliance. Here, the outline of both the cutter and the spindle is hexagonal, and the length of the cutter holder is 6 mm. As shown in Figure 6C, for this assembly task, we divide the whole collaboration into 4 stages in time sequence: 1) Remove the cutter (unsharpened) from the A.T.C system (from 0 to 7 s); 2) Transfer the cutter tool and gradually approach the spindle (from 7 to 21 s); 3) Align the cutter to the spindle (from 21 to 32 s); and 4) Insert the cutter into the spindle to complete the whole cutter replacement process (from 32 to 40 s), which has never been done by other soft robots. We note that stages 3 and 4 involve a straight motion as the Soft Co‐bot needs to insert the cutter into the spindle after careful alignment. This is undoubtedly difficult as the motions of soft robots are usually bending and morphing. We solve this problem by taking advantage of the compliance of the soft materials. More specifically, during alignment at 32 s in Figure 6C, we manually control the cutter to reside against the inner socket of the spindle, and we then forward drag the lower half section and push the cutter into the spindle by squeezing the soft actuator body. Due to the excellent compatibility of the Soft Co‐bot, the interaction forces during these squeezes are quite small and will not damage the cutter and the spindle compared to rigid robots. This shows that the intrinsic softness of the Soft Co‐bot greatly reduces the interaction forces and the effects of mechanical hard contact during task execution, minimizing the possibility of workpiece damage and task failure. We finally demonstrate that the Soft Co‐bot could repeat the cutter replacement process independently (Movie S8, Supporting Information) with a high success rate of up to 90% for 30‐time trials (inset in Figure 6E).

## 3 Conclusion

Although there is clearly excellent environmental and uncertainty compatibility, the performance of this proposed Soft Co‐bot still lags behind the advanced rigid collaborative robots, in terms of precision, speed, etc. This is mainly due to the limitations of soft materials such as hyper‐elasticity, causing a lower response accuracy compared to rigid motor‐driven mechanisms. Local and on‐demand stiffness enhancement could be explored to improve toward this end. Second, the successful rate of this drag teaching control strategy will decrease when the soft robot becomes larger in size or more complex in structure (such as a three‐section assembly instead of two in this work), which could be only improved via a completely novel soft robot structure design and higher performance servo motors. Third, the motion space of this Soft Co‐bot is limited in a parabolic zone in 2D if not considering the turntable, which could be attributed to a one‐sided pneumatic and cable actuation. Therefore, an omni‐directional pneumatic network structure with more equally spaced cables could be used as a more advanced version of this work, in order to enlarge the working space. We note that the robots in this work serve as the first soft collaborative robots with intuitive human drag teaching and could spark future innovations.

In summary, we investigate collaborative interaction between humans and soft robots by proposing a Soft Co‐bot with easy programming and high motion precision. This Soft Co‐bot consists of a soft continuum robot under antagonistic pneumatic and cable excitations, which possesses a simple physical model as a one‐to‐one mapping existing from cable length to robot deformation, and also a precise, low‐hysteresis, well‐damped transient response and excellent motion repeatability due to antagonistic stiffening. More importantly, we have developed a drag tracking and programming system based on a cable tension sensing mechanism and a clear relationship between tension and human drag. Therefore, the two main steps involved in Co‐bots contact‐based drag teaching and high‐precision repetition are both achieved in our proposed design. It is shown to perfectly and gently finish various fine industrial positioning and assembling tasks as never could by all previous soft robots and only possible by those rigid Co‐bots, opening a promising pathway to the wide industrial deployment of soft robots. In addition, the excellent biocompatibility of the Soft Co‐bot has unleashed them from stringent confinement compared to rigid robots, removing segregation, gentler mechanical assembly, and safer interactions.

## 4 Experimental Section

### Fabrication of the Proposed Soft Co‐bot

The soft robot body was fabricated by silicone casting and was divided into 2 parts, the top layer with 30A hardness and the bottom layer with 50A hardness. All molds were fabricated by 3D printing with resin material, in three main steps. First, different silicone solutions were poured into the corresponding molds and cured at 80 °C for 3 h (Figure S1A, Supporting Information). The cured top and bottom layers were carefully removed from the molds and were bonded together (Figure S1B, Supporting Information). Then, frames 2 were inserted into the slots at the two sides of the middle net airbags and frame 1 was inserted into the other net airbags. The connection between flexible continuum sections and the steel frames was sealed with glue (Loctite 416). And finally, different parts and cables (No.72 nylon rope, 1 mm diameter, Zhejiang Jingyi Technology Co.) were mounted on the robot body to form the final soft continuum actuator (Figure S1C,D, Supporting Information). Two servo motors (LD‐3015MG Servo, Hiwonder Technology.) were utilized to actuate the 2 sections of the Soft Co‐bot.

### Performance Characterization

The dynamic response curves and repeatability errors of antagonistic soft actuators were measured by a thin film pressure sensor which was used as a curvature sensor (YD‐SF23‐600 Flexible sensors, Suzhou Leanstar Electronic Technology Co.). The cable tension was measured by tension sensors (JZHL‐M1 tension sensors, Kino Sensors Co.). The initial images for documenting positioning errors were obtained by a high‐speed camera (Canon, EOS R3), and a bracket (VCT‐880, YUNTENG Co.) was used to align the camera's posture to keep it parallel to different measured surfaces for obtaining accurate visual data, then, the positioning errors were acquired by processing the pixel points on the initial images in MATLAB (R2023a).

### Drag Teaching Control Flow

The drag teaching of the Soft Co‐bot was based on feedback from the tension sensors and servos. The robot body was a two‐section antagonistic soft continuum actuator with two cables and two tension sensors. Section 1 (the upper half section) of the Soft Co‐bot was first controlled at a fixed bending angle (e.g., 0 degrees in Figure S20A, Supporting Information). Then, Section 2 (the lower half section) of the Co‐bot was controlled to move and the readout of tension sensor 1 was monitored. After that, the bending angle of Section 1 of the Soft Co‐bot was changed  and the above operation was repeated, and finally, the variation range of tension in cable 1 when the Section 1 of the Co‐bot was at different bending angles could be obtained (Figure S20C, Supporting Information). Similarly,   the variation range of tension in cable 2 when the Section 2 of the Co‐bot was at different bending angles could also be obtained (Figure S20D, Supporting Information).

Based on these experimental results, a bending angle of *∂*, the measured cable tension as *T_∂_*, forward critical drag tension as *T*_∂‐Forward_ and reverse critical drag as *T*_∂‐Reverse_ was defined. The collaboration control logic can be expressed as:

(4) $$\mathrm{Forward}\;\mathrm{bending}:T_{\partial }>T_{\partial -\mathrm{Forward}}$$

(5) $$\mathrm{Reverse}\;\mathrm{bending}:T_{\partial }<T_{\partial -\mathrm{Reverse}}$$

The drag teaching process starts with the user's hand dragging the Soft Co‐bot, and the sensors system (including both tension sensors and potentiometers in servos) transforms this external disturbance into output (including both cable tension change and servo rotation angles) and sends the sensing data to the control center in real‐time. Based on the proposed control algorithm, the control center performs decision‐making on the drag direction and sends the control command (control input) to the target system. The controller takes ≈0.1 s to complete the drag judgment, then the servo actuates the Soft Co‐bot moves to a new state and then proceeds to the next cycle (Figure 4D).

### Collaborative Control of the Turntable

The turntable was utilized to extend the motion of the Soft Co‐bot from 2D plane to 3D space for more application scenarios (Figure S21, Supporting Information). For the achievement of the collaborative control of turntable rotation by dragging the Soft Co‐bot, 2 thin force gauges on the bottom of the robot body to monitor the squeezing force on the bottom sides of the Co‐bot were placed (Figure S22A, Supporting Information). The readouts of two force gauges will change when there was an external disturbance applied to the sides of the Soft Co‐bot (Figure S22B, Supporting Information), and the change was related to the direction of disturbances. Based on these, the turntable rotation by dragging the sides of the Soft Co‐bot and monitoring the changes in the readouts of two force gauges was collaboratively controlled (Figure S22C, Supporting Information).

## Conflict of Interest

The authors declare no conflict of interests.

## Supporting information

Supporting Information

Supplemental Movie 1

Supplemental Movie 2

Supplemental Movie 3

Supplemental Movie 4

Supplemental Movie 5

Supplemental Movie 6

Supplemental Movie 7

Supplemental Movie 8

## Data Availability

The data that support the findings of this study are available from the corresponding author upon reasonable request.
