# Supplementary material for: A Soft Collaborative Robot for Contact‐based Intuitive Human Drag Teaching
Source: Adv Sci (Weinh). 2024 Apr 22;11(24):2308835. doi: 10.1002/advs.202308835 (PMC11200028; doi:10.1002/advs.202308835)
Supplement: Supplementary file 1 — Supporting Information [file ADVS-11-2308835-s001.pdf]

ADVANCED SCIENCE
Open Access

Supporting Information

for *Adv. Sci.*, DOI 10.1002/advs.202308835

A Soft Collaborative Robot for Contact-based Intuitive Human Drag Teaching

Shoulu Gong, Wenbo Li, Jiahao Wu, Bohan Feng, Zhiran Yi, Xinyu Guo, Wenming Zhang* and Lei Shao**

Supporting Information

A Soft Collaborative Robot for Contact-based Intuitive Human Drag Teaching

Shoulu Gong, Wenbo Li, Jiahao Wu, Bohan Feng, Zhiran Yi, Xinyu Guo, Wenming Zhang* and Lei Shao**

*Corresponding author. Email: wenboli@tongji.edu.cn, wenmingz@sjtu.edu.cn, lei.shao@sjtu.edu.cn

The supporting information includes:

Supplementary Text 1 to Text 10

Figure S1 to Figure S22

Supplementary Table S1 to Table S3

Legends for Movie S1 to S8

Movie S1: Segment actuation independence testing after stiffness enhancement.

Movie S2: High-precision repetitive motion of the Soft Co-bot based on open-loop control.

Movie S3: Repetitive balloon contacting with a needle.

Movie S4: Safe obstacle collision during repetition.

Movie S5: Drag teaching for high-precision pin latching operations.

Movie S6: Soft Co-bot for Bolt positioning.

Movie S7: Soft Co-bot for electronics assembly.

Movie S8: Soft Co-bot for cutter replacement.

Supplementary Text 1: Fabrication process of the soft continuum actuator

Supplementary Fig. s1 shows the fabrication and assembly process of the soft continuum actuator. The actuator body consists of a top layer (the bending layer, Ecoflex-30, 30A hardness) and a bottom layer (the constrained layer, Ecoflex-50, 50A hardness), both of which are fabricated by molding process (Supplementary Fig. s1a). In particular, one paper layer is embedded into the bootom layer to constrain its stretch deformaiton. Then, we mount these two layers together to form the continuum actuator body (Supplementary Fig. s1b). All other parts are fabricated by stereolithography (SLA) with resin materials. After that, we install different types of frames (with the frames 2 are inserted into the slots at the two sides of the middle net-airbags and the frames 1 are inserted into the other net-airbags) into the continuum actuator body in sequence for cables guidance, followed by other parts including the top part, the connection, and the bottom part. We finally install the soft continuum actuator to the base platform (Supplementary Fig. s1c). In this work, two servo motors are used to construct the actuation platform and are connected to the soft continuum actuator via cables. Both servo motors are controlled independently of each other, where the servo 1 is utilized to actuate the upper half section of the soft continuum actuaor and the servo 2 is utilized to actuate the lower half section (Supplementary Fig. s1d).

In this work, two servos are utilized to control the motion of the two-section soft continuum actuator after pre-inflation, we can control its motion by actuating two servos to reel in or reel out the cables. Supplementary Fig. s2 shows the detailed actuation process of the proposed pneumatic-cable driven antagonistic actuation strategy. Taking a single actuator as an example, we first increase its inner pressure via a single check valve, and the actuator will be pneumatically actuated to reach a bending state. Then, we can control the actuator to return to its initial state or a reverse bending state by reeling in the cable.

Supplementary Text 2: Deformation simulation of the soft actuator

A deformation simulation is performed to analyze the motion states of the soft bending actuator with separate narrow slots under pressure. The finite element method (FEM simulation is conducted using ABAQUS/Standard (Simulia, Dassault Systems, 6.14). The geometry and material property parameters can be found in Supplementary Table S1. The soft bending actuator is modeled with 3D solid elements (C3D8RH) and four hyper elastic models are used to simulate the constitutive behavior of Ecoflex-30 and Ecoflex-50, the detailed model parameters are shown in Supplementary Table S2. The model contains a total of 3615 elements. Assembling the soft bending actuator by creating an instance and connecting the bending layer and bottom layer by creating a face-to-face constraint. For the boundary condition, the bottom of the actuator is fixed. There are 2 steps beside the initial step to simulate the motion of a soft bending actuator, including a step-G for gravity loading and a step-P for air pressure loading. All these steps are used a direct, full Newton solver."

Supplementary Text 3: Stiffness enhancement via antagonistic actuation

Soft robots have shown better morphological capabilities than traditional rigid robots owning to their compliant body structures. However, its compliant nature leads to low output force and poor payload capability, limiting its application scenarios. Therefore, the on-demand stiffness enhancing technology is a key milestone in the field of soft robots. Towards this end, antagonistic muscles allow humans to adjust the stiffness of their limbs to achieve sophisticated motion trajectories and control when handling objects via precise control of interaction forces (Supplementary Fig. s4a). In this work, pneumatic and cable-driven antagonistic actuation strategy is designed for stiffness control. Based on antagonistic actuation, this soft actuator can realize fast stiffness enhancement via adjusting its pre-inflation pressure (within one second). The essence of the stiffness enhancement of the soft actuator is an increase in its potential energy by air inflation. The potential energy of the soft actuator at different pre-inflation pressures and bending angles can be calculated based on the beam bending theory. The total energy U of the actuator can be expressed as:

$$U = U_{bending} + U_{strain} \tag{1}$$

In which, U_{bending} means the bending energy and U_{strain} means the strain energy. The bending energy and strain energy of the actuator can be calculated based on the beam bending energy analysis as:

$$U_0 = \int_0^L \frac{EI\kappa^2}{2} dl + \int_0^L \frac{\varepsilon^2 EA}{2} dl \tag{2}$$

$$U_1 = \int_0^L \frac{EI\kappa^2}{2} dl + \int_0^L \frac{\varepsilon^2 EA}{2} dl + \Delta PV \tag{3}$$

In which E is the elastic modulus of the silicone, L and A are the length and cross-sectional area of the actuator, V is the volume of the chamber, I is the moment of inertia and ε is the strain. U_0 is the total energy of the actuator before stiffness enhancement (before increasing the pre-inflation pressure), where the first and second term represents the bending energy and represents the bending and stretching energy in the soft actuator. And U_1 is the total energy of the actuator after stiffness enhancement (after increasing the pre-inflation pressure). ΔPV is the increased energy due to pneumatic work.

Supplementary Fig. s4b shows the detailed stiffness enhancement process of the soft actuator via antagonistic effect. When the pre-inflation pressure of the actuator is 40 kPa,we assume the resultant moment is M_1. Based on the beam theory, the slope θ_B of beam under an equivalent concentrated force F can be expressed as:

$$\theta_B = -\frac{FL^2}{2EI} \tag{4}$$

In which, E is the elastic modulus of the silicone, L and I are the length and the cross-sectional moment of inertia. The equivalent concentrated force F can be expressed as:

$$F = F_{Penumatic} - F_1 \tag{5}$$

In which, $F_{\text{pneumatic}}$ is the output force of the actuator with a pressure of 40 kPa, and F_1 is the cable pulling force, while $F_{\text{pneumatic}}$ and F_1 are antagonistic. When we increase the pre-inflation pressure of the actuator to 65 kPa, the cable pulling force will increase to F_2 to maintain this equilibrium state and the antagonistic effect is enhanced, resulting in a stiffness enhancement. For clearer verification, we also characterize the load capacity of the soft actuator after stiffness enhancement. As shown in Supplementary Fig. s4c, we first demonstrated that a soft gripper (with two antagonistic soft actuators) can grasp 1500-gram objects (more than 15 times its own weight), and then showed that the actuator can support an end-load of 200-gram in a horizontal state (more than twice its own weight).

Supplementary Text 4: Experimental verification of the pre-inflation pressure

Most soft actuators often show poor response performance (poor repeatability, high hysteresis and susceptibility to external disturbances) due to their inherent flexibility and materials limitations. Suitable stiffness enhancement can effectively improve the response performance of soft robots without affecting their kinematic capabilities. However, some traditional stiffness enhancement methods for soft robots (e.g., granular jamming, layer jamming, shape memory alloys or other variable stiffness materials for soft robots) are achieved by increasing the structural stiffness or changing the material properties, which limits the kinematic capabilities of soft robots in high stiffness states. The principle of the proposed bionic stiffness enhancement strategy for soft robots is the pneumatic-cable antagonistic effect, which has a small impact on the kinematic capabilities of soft robots within a certain stiffness enhancement range.

For choosing a suitable pre-inflation pressure, there are two main components need to be considered. One is the motion range (in particular, the range of forward bending angles) of soft actuator, which is because the maximum forward bending angle of the actuator is determined by the pre-inflation pressure (complete positive correlation), which directly determines the working space of the Soft Co-bot. Supplementary Fig. s5 shows the measured bending angles of a single soft actuator (without the cable for pulling) as a function of air pressure. It's clear that the pre-inflation pressure needs to be larger than 60 kPa to ensure the maximum forward bending angle of the soft actuator can exceed 100 degrees, thus guaranteeing a sufficient working space for the Soft Co-bot. The other component to be considered is the appropriate stiffness enhancement effect after increasing the pre-inflation pressure of the soft actuator. We know that antagonistically arranged muscles allow animals and humans to adjust the stiffness of their limbs to achieve sophisticated motion trajectories and precise control of interaction forces when handling objects; however, when the muscles are tensed to a certain degree (with relatively high stiffness), humans are not able to achieve precise control over the accuracy of the movement and the force with which it is performed. The proposed pneumatic-cable antagonistic pairs are similar to the antagonistically arranged muscles, where excessive stiffness enhancement would hinder the kinematic capabilities of soft robots and cause additional energy consumption due to the cables requiring a larger force to pull the robots. Hence, we performed some experiments to characterize the relationship between the stiffness enhancement effect and the magnitude of the pre-inflation pressure. Supplementary Fig s6 shows the cable pulling force (measured by a push force meter: HP-2, Wenzhou Haibao Instrument Co., Ltd.) of the antagonistic actuator as a function of pre-inflation pressure at different fixed bending, including 50 degrees, 70 degrees

and 90 degrees. It is obvious that the cable pulling force of the antagonistic actuator increases with increasing pre-inflation pressure, which represents an increased pneumatic-cable antagonistic effect, meaning a stiffness enhancement of the soft actuator. Taking the antagonistic actuator with a bending angle of 90 degrees (sufficient for the working space requirements of the Soft Co-bot) as an example.

As shown in Supplementary Fig. s6c, the antagonistic effect increases with increasing pre-inflation pressure, and the increment is particularly pronounced (with a relatively large slope of 0.12 N/kPa) before the pre-inflation pressure reaches 80 kPa. After that, the enhanced antagonistic effect is no longer as effective (with a smaller slope of 0.0636 N/kPa) with increasing pre-inflation to 100 kPa. Moreover, a pre-inflation pressure of 100 kPa will cause more energy consumption and harm the kinematic capabilities and intrinsic softness of the Soft Co-bot. So, we finally chose the pre-inflated pneumatic pressure at 80 kPa for the Soft Co-bot to ensure enough stiffness enhancement and working space for movement without compromising its kinematic capabilities.”

Supplementary Text 5: Analysis and testing of the improved response performance

In nature, high dexterity and stiffness control can be achieved simultaneously and without the need for an underlying skeletal structure. For example, the octopus is a mollusk, which can move its flexible arms without a bone in its body. Each octopus arm can be individually controlled to follow motion trajectories, can be squeezed to fit through narrow passages and can stiffen to conduct tasks such as catching prey or walking on the seabed. The moderate stiffness enhancement of the soft actuator through antagonistic effects not only does not affect their flexibility, but also effectively improves their response performance. On the one hand, the antagonistic effect partially eliminates the influence of soft material hyper elasticity on the robot motion and improves its dynamic response performance. On the other hand, the antagonistic effect appropriately increases the stiffness of the joints and improves the motion repeatability by choosing an appropriate pre-inflation pressure.

To characterize the enhanced response performance of the antagonistic actuator with a pre-inflation pressure of 80 kPa, we utilized a thin film pressure sensor (YD-SF23-600 Flexible sensors, Suzhou Leanstar Electronic Technology Co.) to record the bending angles of the actuator at a sampling frequency of 50 Hz (shown in Supplementary Fig.s7). We separately characterized the response performance of pneumatic soft actuators, cable-driven soft actuators and antagonistic soft actuators (shown in Supplementary Fig. s8a-8c, from left to right).

For the dynamic response tests, to ensure the fairness of the experimental results, we set a uniform actuation time of 0.5 seconds, a pressure of 80 kPa for the pneumatic actuation (same as the pre-inflation pressure), and a servo rotation angle of 130 degrees. As shown in Supplementary Figs. s8d-8f, the antagonistic soft actuator shows better dynamic response, including smaller overshoots, smaller steady-state error and faster recovery times, which is mainly due to the reason that the pneumatic-cable driven antagonistic effect greatly improves the hyper elastic property of the soft silicone material and increases the motion stiffness. In terms of the response time, the pneumatic soft actuators show the fastest response time (As shown in Supplementary Fig. s8g), but pneumatic actuators usually have a negligible response hysteresis (which is evident in Supplementary Fig. s8d) and it is difficult to balance the actuation speed and control accuracy. The antagonistic actuators (Supplementary Fig. s8i) show a faster response time than the cable-driven actuators (Supplementary Fig. s8h), which is due to that the test scenario is a bending motion process, and the pre-inflation pressure increases the potential energy of the antagonistic actuators, which effectively improves its response speed. Supplementary Figs. s8j-8l show the motion repeatability tests for 50 cycles of pneumatic actuators, cable-driven actuators and antagonistic actuators, from left to right. The antagonistic

soft actuators show the best motion repeatability with an average error less than 0.8% (calculation: absolute (measured bending angle – theoretical bending angle) / theoretical bending angle), which is mainly attributed to the appropriate increase in stiffness caused by the antagonistic effect, leading to improved stability of the actuator. Based on the above experiments, we have verified that a pre-inflation pressure of 80 kPa can effectively improve the response performance of the antagonistic actuator and does not affect its kinematic capabilities, which still has a large bending range from -100 degrees to 100 degrees."

Supplementary Text 6: Geometric design of the actuator body and Frame 2

In this work, we propose a kinematic modeling approach based on the geometric relationship between the cable length variation and the bending angle of the antagonistic actuator. This modeling approach is based on a piece constant curvature (PCC) assumption. Taking a single antagonistic soft actuator as an example, bounded by the position of Frame 2 (the dotted line in Supplementary Fig. s4a), we divided the soft actuator into two continuous parts (the solid lines in Supplementary Fig. s4a) and assume that each part is an arc of certain curvature during the bending process. To make the actual bending behavior of the actuator more consistent with the assumptions, we first increase the thickness of the bottom layer of the actuator and integrate a non-stretchable constraining layer inside it to limit its tensile deformation under pressure. Secondly, we increase the wall thickness of the air chambers, reduce their number and distribute them only in the middle of the actuator to optimize the deformation behavior of the actuator under different pressures. After validation with FEM analysis, we determine the final geometric dimensions of the soft actuator (shown in Supplementary Table S1). Supplementary Fig. s4b shows the actual bending behaviors of the actuator under different actuation states, and it can be observed that its bending behaviors closely fit the constant curvature assumption. Which greatly improves the accuracy of the kinematic model.

We note that another key point to maintain this accurate geometric relationship is to always keep the cable straight during the whole morphing process. To achieve this goal, we design a special Frame 2 with a narrow opening to prevent contact between the cable and actuator body. Supplementary Fig. s10 shows the cross-sectional view of the actuator in both bending and reverse bending states. The narrow opening in Frame 2 divides the cable into two parts, minimizing the friction of the cable during the entire motion process (the cable is only in contact with the narror opening in Frame 2) and eliminating hysteresis that commonly exisits in the cyclic motion of cable-driven robots (Fig. 2D).

Here, we consider two critical conditions of the actuator during one single motion cycle: the maximum bending state and the maximum reverse bending state. Supplementary Fig. s10a shows the schematic of the maximum bending state, h is the height of the narrow opening, t = 9.2 mm (thickness of the constrained layer), d_1 is the minimum distance between the cable (the blue line) and surface 1. Based on the geometric relationship between these parameters, the minimum distance d_1 can be expressed as:

$$d_1 = (R_3 + t + h)\cos(\partial_3 / 2) - R_3 - t \quad (6)$$

Here, R_3 denotes the equivalent radius, which can be calculated using the following equation:

$$R_3 = \frac{L_3}{\partial_3} \tag{7}$$

To always keep the cable straight (i.e., there is no contact between the cable and surface 1), d_1 should be greater than zero:

$$(R_3 + t + h)\cos(\partial_3 / 2) - R_3 - t \geq 0 \tag{8}$$

$$h \geq \frac{R_3 + t}{\cos(\partial_3 / 2)} \text{-} R_3 - t \tag{9}$$

Considering our application scenarios, we set the maximum of ∂_3 to 45 degrees (90 degrees for a complete actuator). In this critical condition, h should be greater than 6.422 *mm*, which is the lower limit of the position for the narrow opening.

In the reverse bending state shown in Supplementary Fig. s10b, d_2 is the maximum distance between the cable (the yellow line) and constrained layer. Similarly, h_2 can be expressed by other geometric parameters, as shown in the following equation:

$$d_2 = R_2 - (R_2 - t - h)\cos(\partial_2 / 2) - t \tag{10}$$

$$R_2 = \frac{L_2}{\partial_2} \tag{11}$$

Again, to always keep the cable straight (i.e., there is no contact between the cable and surfae 2), d_2 should be smaller than the height of Frame 2 (18.5 *mm*)..

$$R_2 - (R_2 - t - h)\cos(\partial_2 / 2) - t \leq 18.5 \tag{12}$$

$$h \leq \frac{18.5 + t - R_2}{\cos(\partial_2 / 2)} + R_2 - t \tag{13}$$

Considering our application scenarios, we set the maximum of ∂_2 to 45 degrees (- 90 degrees for a complete actuator). In this critical condition, h should be smaller than 15.118 *mm*, which is the upper limit of the position for the narrow opening.

So, we choose h = 10 *mm* for Frame 2.

Supplementary Text 7: Kinematic modeling

Supplementary Fig. s11 shows the kinematic analysis of the antagonistic two-section soft cotinuum actuator. Two servo motors are utilized to wound two cables through reels and to control the two sections of the soft continuum actuator respectively. We first inflate the soft continuum actuator through a single check valve to increase its inner pressure. However, the actuator could be maintained as no bending by proper cable pulling due to the antagnistic effect. Then, we control servo 2 to reel out the Cable 2, and the Section 2 (the lower half section) of the soft continuum actuator will reach a bending state. We can also control servo 1 to reel in the Cable 1, and the Section 1 (the upper half section) will reach a reverse bending state. Thus, the bending angles of two sections in the soft continuum actuator can be controlled by the rotation angles of servos independently. Compared to analyzing the hyperelastic deformation of soft actuators, this kinematic modeling approach is much simpler and more accurate as it based on the geometric relationship between the cable length variations (which also solves for the servo rotation angles) and the actuator bending angles. We set the neutral state (Supplementary Fig. s11(i)) as a reference. The kinematic model of the bending state (Supplementary Fig. s11(ii)) can be derived as below:

$$L_{31} = 2(R_3 + t + h)\sin(\partial_3 / 2) \tag{14}$$

$$\Delta L = 2(\frac{L_3}{\partial_3} + t + h)\sin(\partial_3 / 2) - L_3 \tag{15}$$

$$w = \Delta L / r \tag{16}$$

In which, ∂_3 is the bending angle of a portion of the lower half section, $\triangle L$ is the length variation of the Cable 1, w represents the servo rotation angle, r is the radius of the spool and $h + t$ is the distance between the bottom of the actuator and the small opening of Frame 2. Similarly, the kinematic model of the reverse bending state (Supplementary Fig. s11(iii)) can also be derived as below:

$$L_{21} = 2(R_2 - t - h)\sin(\partial_2 / 2) \tag{17}$$

$$\Delta L = L_2 - 2(\frac{L_2}{\partial_2} - h - t)\sin(\partial_2 / 2) \tag{18}$$

$$w = \Delta L / r \tag{19}$$

Here, ∂_2 is the reverse bending angle of the upper half section, $\triangle L$ is the length variation of the Cable 2. To verify this proposed kinematic model, we also measure the bending angles of the actuator at different servo rotation angles (from -120 degrees to 120 degrees) within one motion cycle. Supplementary Fig. s12 shows the measured actuator bending angles, and errors between theory and experiments as a function of servo rotation angles. The mean error is less

than 2 degrees, showing that the proposed kinematic modeling approach is sufficiently accurate.

For this two-section soft continuum actuator, we can also easily derive its end tip position and posture at different motion commands, thus realizing high-precision open-loop control of the continuum actuator. Fig. 2G shows the D-H modeling parameters. We set the bending angles of the two sections as ∂_1 and ∂_2, and the length of connection part as *d*. Here, X_0OY_0 is the base coordinate. X_iOY_i is the coordinate on different joints, a_i is the distance between joints, and θ_i is the rotation angles between different coordinates. The transition matrix from coordinate i to i+1 can be expressed as:

$$T_{i+1}^{i}(\theta_{i+1}) = \begin{bmatrix} \cos\theta_{i+1} & -\sin\theta_{i+1} & \alpha_{i+1}\cos\theta_{i+1} \\ \sin\theta_{i+1} & \cos\theta_{i+1} & \alpha_{i+1}\sin\theta_{i+1} \\ 0 & 0 & 1 \end{bmatrix} \quad (20)$$

The position of the robotic end-tip (x_0, y_0) in the base coordinate X_0OY_0 can be calculated based on the position (x_4, y_4) on its own coordinate X_4OY_4, and the total transition matrix can be expressed as:

$$\begin{bmatrix} x_0 \\ y_0 \\ 1 \end{bmatrix} = \prod_{i=0}^{3} T_{i+1}^{i}(\theta_{i+1}) \begin{bmatrix} x_4 \\ y_4 \\ 1 \end{bmatrix} \quad (21)$$

The posture of the robotic end-tip with respect to the base coordinate can be expressed as:

$$\partial = \partial_2 - \partial_1 \quad (22)$$

Supplementary Fig. s13 shows the theoretical working space of the soft continuum actuator. Considering the weight of the actuator itself, we set the range of ∂_1 as [-90 degrees, 60 degrees] and the range of ∂_2 as [-90 degrees, 90 degrees]. We also show several motion states of the soft continuum actuator corresponding to specific points in its working space.

Supplementary Text 8: Precise control of the Soft Co-bot after stiffness enhancement

The proper stiffness enhancement improves the motion accuracy of this two-section soft continuum robot during open-loop control, which is mainly attributed to the enhanced mechanical stability. For example, when we only give motion commands to Servo 2, we expect only the lower half section (Section 2) of the continuum actuator to move, with the motion state of the upper half section (Section 1) undisturbed. However, for a soft continuum actuator with a low stiffness (low pre-inflation pressure, the left one in Supplementary Fig. s14b), when we control the Servo 2 to rotate from 0 degree to -100 degree, both two sections of the continuum actuator move, which is due to the fact that the Section 1 of the continuum actuator has less stiffness and is unable to resist the inertial forces caused by Cable 2 during the pulling process. But when we increase the stiffness of the continuum actuator (with a larger pre-inflation pressure of 80 kPa, the right one in Supplementary Fig. s14b), the bending state of its Section 1 will not be disturbed by the motion of its Section 2, which greatly improves the accuracy of open-loop motion control.

As shown in Supplementary Fig. s14a, we designed a pin latching experiment to characterize the control accuracy and repeatability of open-loop control. In the first one, we repeatedly control the robotic end-tip through the Hole 6 within 1.5 seconds by actuating only the lower half section (Supplementary Fig. s14c), and the yellow line represents the motion path. Similarly, we repeatedly control the robotic end tip through the Hole 7 within 2 seconds by actuating only the upper half section (Supplementary Fig. s14d and Supplementary Movie 1). Supplementary Fig. s14e and s14f show the measured pin center positions after 15 repetitive pin latching operations for Hole 6 and Hole 7, respectively. The blue dotted line represents the outline of the hole. In both experiments, the soft robot showed extremely high positioning accuracy and motion repeatability. Also in the Supplementary Movie 1, it can be visualized that when we control only one section of the continuum actuator in motion, the bending state of the other section is undisturbed.

We also tested the positioning accuracy and motion repeatability of the soft continuum actuator under different end loads. As shown in Supplementary Fig.s15a, a laser pointer is added to the end-tip of the Soft Co-bot to measure its motion repeatability. Here, the human worker drags the Soft Co-bot to make the laser hit the target point, and the dragging instructions for repeatability tests under different end loads are same. Then, the Soft Co-bot repeats this dragging-programmed task with differnet end loads, including 0 grams, 20 grams, 50 grams and 100 grams. Supplementary Fig. s15b shows the measured motion repeatability of the Soft Co-bot under different end loads, showing a large deviation between the actual motion positions

and the expected positions of the Soft Co-bot when the end loads exceed 50 grams. The deviation is due to the softness of the material and the large force arm, which becomes larger with increasing end loads. We also note that the positioning errors of the Soft Co-bot during different motion cycles are very consistent with a fixed end load, showing a high motion repeatability with a fixed deviation. In our subsequent research, it is possible to obtain an accurate relationship between end loads and motion deviations of the Soft Co-bot based on extensive datasets and machine learning methods, and to improve the motion accuracy of the Soft Co-bot under high loads via actuation compensation.

In the point to point tracking experiment (as shown in Supplementary Fig. s16), we only input a sequence of control commands to Servo 2 to actuate the lower half section of the soft continuum actuator and record the position of its end-tip after each movement. Supplementary Fig. s16a shows the comparison between the theoretical and experimental results with an average error of 1.5% (absolute position offset / body length). Then, we add a 100 gram load to the end-tip of the soft continuum actuator and repeat the point to point tracking experiment. Supplementary Fig. s16b shows the comparison between the theoretical and experimental results with an average error of 2.2% (absolute position offset / body length). The experimental results match well with our theoretical model, demonstrating the reliability of the soft continuum actuator to perform precise tasks based on open-loop control.

Explanation of three dimensional coordinate errors: The absolute positioning error and repetitive positioning error are important metrics for control accuracy and repeatability of the Soft Co-bot. In the manuscript, the position errors mean the actual motion position and desired motion position of the end marker point of Soft Co-bot. Therefore, although the motion of the Soft Co-bot is in three-dimensional coordinates, the experimental characterizations we performed, such as the bolt assembly, electronics assembly, cutter replacement, etc., depended on the alignment in the 2D work plane. So, we demonstrated the high accuracy and repeatability of the soft collaborative robot by showing the positioning errors in the 2D work plane, including Figure 1C, Figure 3B, Figure 5C, Figure 6D, Figure 6E, Supplementary Fig. s14e and s14f. During the image data processing, the diameter of the end marker point is revised to 0.1 mm in MATLAB to eliminate the effect of its size on positioning errors.

Supplementary Text 9: Design of the collaborative drag teaching system

Soft robots usually possess a poor mechical stability and are prone to deformation when subjected to external forces. But in this work, the proposd antagonistic actuation stratregy endows the soft continuum robot with stiffness enhancement, thus improving its stability under external dragging. As shown in Supplementary Fig. s17, in this experiment, a force gauge is used to apply external disturbances to different positions of the antagonistic soft continuum robot. An ultrasonic distance sensor is used to measure the passive movement of the robot body under different external disturbances. Supplementary Fig. s17b – s17e show the measured passive movement of different positions on robot body under various external disturbances. The experimental results show that the antagonistic soft continuum robot can resist an external force of at least 2.5 Newtons without significant passive movement.

In addition, it is also a challenge to accurately recognize the user's drag direction and execute the corresponding motion commands in a cooperative manner. For the antagonistic soft continuum robot, we note that the cable tension of the robot will change when the robot encounters external disturbances. So, we choose to construct the dragging control algorithm based on the tension variations of two cables in the soft continuum robot. To achieve this goal, we design a cable tension measurement system for the antagonistic soft continuum robot, which contains two tension sensors and data acquisitions. As shown in Supplementary Fig. s18, two tension sensors are placed on each side to detect the tension variation in the two cables, while the two cables are guided by pulleys to contact the tension sensors and tied to the servo motors. This cables-sensors-servos arrangement allows the cables to be always tightened, which improves the tension measurement accuracy.

Taking one section of the soft continuum actuator as an example, Supplementary Fig. s19a shows the mechanical analysis of the single actuator under different external drags. Here, we define that ∂ is the bending angle of the actuator, M is the bending moment of the actuator caused by inner pressure. T_∂ is the cable tension, and M_T is the equivalent torque of the tension T_∂ at the end of the actuator. When there is no external dragging (Supplementary Fig. s19a(i)), this equilibrium state be expressed as:

$$M_T = M \tag{23}$$

At this point, we set T_∂ as the tension reference value at the bending angle of ∂. When the actuator is under an external disturbance (as shown in Supplementary Fig. s19a(ii), the yellow arrow is the draging direction and F' is the draging force), the initial equilibrium state is broken due to the external torque M' (caused by draging force F') and a new equilibrium state is established, which can be expressed as:

$$M_{T1} = M + M' \tag{24}$$

$$M_{T1} > M_T, T_{\partial 1} > T_\partial \tag{25}$$

In this new equilibrium state, the cable tension is increased to $T_{\partial 1}$ (which is larger than T_∂) and the equivalent torque is M_{T1}, while the dragging direction is defined as forward dragging. Similarly, when the actuator is under an opposite external dragging as shown in Supplementary Fig. s19a(iii), the final equilibrium can be expressed as:

$$M_{T2} = M - M' \tag{26}$$

$$M_{T2} < M_T, T_{\partial 2} < T_\partial \tag{27}$$

In this equilibrium state, the cable tension is decreased to $T_{\partial 2}$ (which is smaller than T_∂), the equivalent torque is M_{T2} and the dragging direction is defined as reverse dragging. To ensure each section of this soft continuum actuator responding to the external dragging and performs correspoinding movements, we set several cable tension intervals for different robot bending angles. Supplementary Fig. s19b shows the measured tension of the cable 2 as a function of time when the lower half section of the soft continuum actuator has a bending angle of 60 degrees. The cable tension is about 1.7 N with no external disturbance (the gray shading as the error bar). In general, when the bending angle of the lower half section equals to ∂ and there is no external disturbance, the measured tension value of cable 2 should be T_∂. When we apply an external drag to the lower half section, the tension value of the cable 2 will change. Here, we set two thresholds, including the forward dragging threshold $T_{\partial\text{-Forward}}$ and the reverse dragging threshold $T_{\partial\text{-Reverse}}$. If the feedback value from the tension sensor is greater than $T_{\partial\text{-Forward}}$, the lower half section of the continuum actuator detects a forward dragging and it will bend forward. Otherwise, if the feedback value of the tension sensor is smaller than $T_{\partial\text{-Reverse}}$, the lower half section detects a reverse drag and it will bend in the reverse direction (Supplementary Fig. s19c).

For the two-section soft continuum actuator, two cables (cable 1 and cable 2) are used to actuate the two sections respectively. So, the cable tension variations will be more complicated due to the fact that the motion of the two segments on the soft continuum actuator are interfering with each other. For accurate cable tension measurement, we first maintain the upper half section (Section 1) of the soft continuum actuator at a bending angle of 0 degree (neutral state, maintain the servo 1 stationary). We then control the lower half section to move from – 80 degrees to 80 degrees and monitor the tension variations of the cable 1. We found that even with the interference of the lower half section movement, the tension value of cable 1 is only slightly change in a certain range (Supplementary Fig. s20a). After that, we change the bending angle of the upper half section and repeat the above test. Supplementary Fig. s20c shows the measured tension variations of cable 1 as a functon of the bending angles of Section 1 (the upper half

section), taking into account the motion of Section 2 (the lower half section), while the grey shading is the error bar. Based on these experimental results, we set two critical lines, including the forward bending threshold for the upper half section to make the judgement of forward dragging (the red dotted line) and the reverse bending threshod for the upper half section to make the judgement of reverse dragging (the green dotted line).

As an example, if the bending angle of the upper half section is ∂ (feedback from the servo system), and the measured tension of cable 1 is T_∂, the decision making logic can be expressed as:

$$\textit{Forward bending}: \; T_\partial > T_{\partial - Forward} \tag{28}$$

$$\textit{Reverse bending}: \; T_\partial < T_{\partial - Reverse} \tag{29}$$

Similar experiments are also performed on the Section 2 (the lower half section) of the soft continuum actuator (Supplementary Fig. s20b and s20d). Combined with the above theoretical analysis and experimental merasurement results, based on the tension signal feedback and servo rotation angle feedback, the Soft Co-bot can not only sense its own motion states, but also judge the direction of dragging, and thus to realize collaborative operation.

Explanation of the sample rate: The sample rate of the collaborative robot represents the amount of external dragging judgements that the Soft Co-bot can complete in one second. A moderate sample rate is benefit for both the drag identification and the motion reliability. On the one hand, if the sample rate is too low, the Soft Co-bot will not be able to recognize the external dragging actions in time; On the other hand, if the sample rate is too high, the teaching motion of the Soft Co-bot under external dragging actions will be too sensitive, which greatly affects its teaching reliability. Meanwhile, considering the processing capability of the Arduino mega board, we finally selected 10 Hz as the sample rate for the Soft Co-bot and verified its validity in the subsequent demonstration.

Supplementary Text 10: Design and collaborative operation of the rotational turntable

As shown in Supplementary Fig. s21a, we design a turntable for this Soft Co-bot, which extends the motion space of the Soft Co-bot from a two-dimensional plane to a three-dimensional space. Supplementary Fig. s21b shows that the Soft Co-bot with a gripper successfully complete the resistor transfer job onto the breadboard. The turntable extends the application scenarios for the Soft Co-bot, allowing the robot to be used for more complex tasks.

We then consider how to achieve collaborative operation with the turntable by dragging the Soft Co-bot. To achieve this goal, we place two thin force gauges on the bottom sides of the Soft Co-bot body (as shown in Supplementary Fig. s22a). Supplementary Fig. s22b shows the force gauges signal feedback during collaborative process. When we drag the end tip of the Soft Co-bot to the right, the sensing value of the force gauge 1 (the blue and left one in Supplementary Fig. s22c) increases significantly. Conversely, when we drag the end tip of the Soft Co-bot to the left, the sensing value of the force gauge 2 (the orange and right one in Supplementary Fig. s22c) increases significantly. This trend is due to the squeezing of the robotic bottom on the two side gauge sensors when the user drags the Co-bot in different directions. Based on this, the collaboration on the rotation motion of the turntable is achieved by direct human dragging.

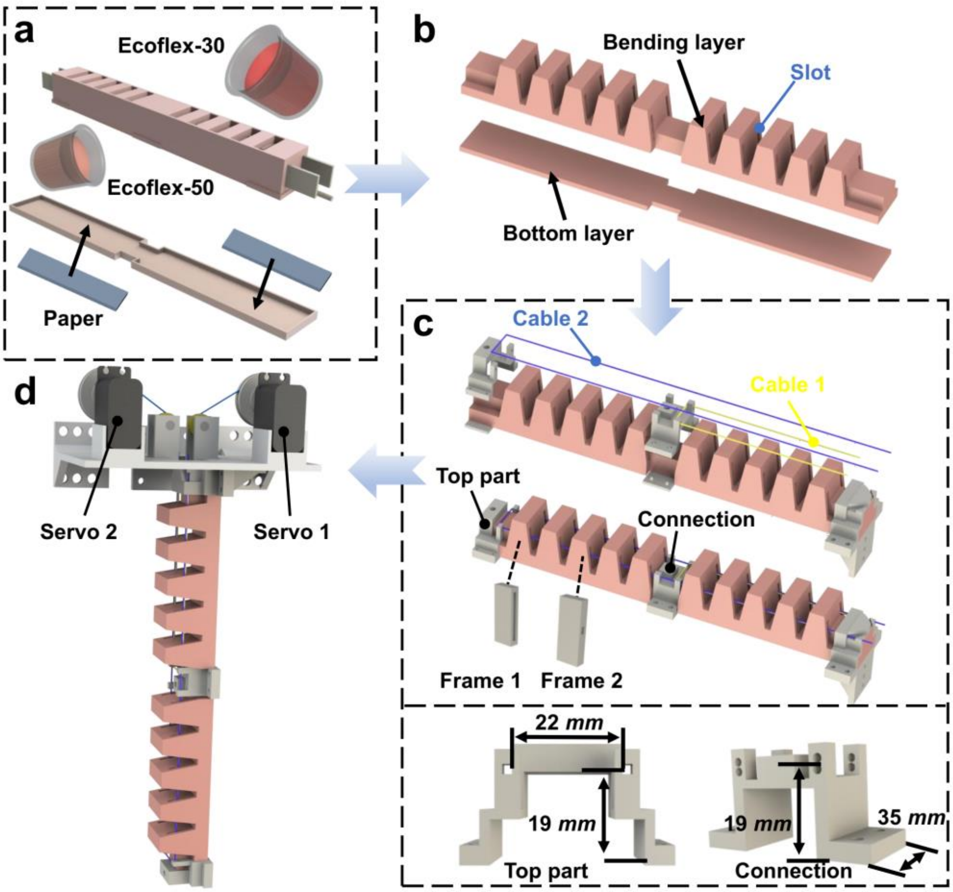

Figure S1: Fabrication and assembly process of the two-section antagonistic soft continuum actuator. (a) Fabrication of the bending layer and the bottom layer of the actuator via molding process. (b) Mount both layers together to form the soft continuum actuator. (c) Schematic of assembling of all parts. (d) Schematic of the two-section soft continuum actuator.

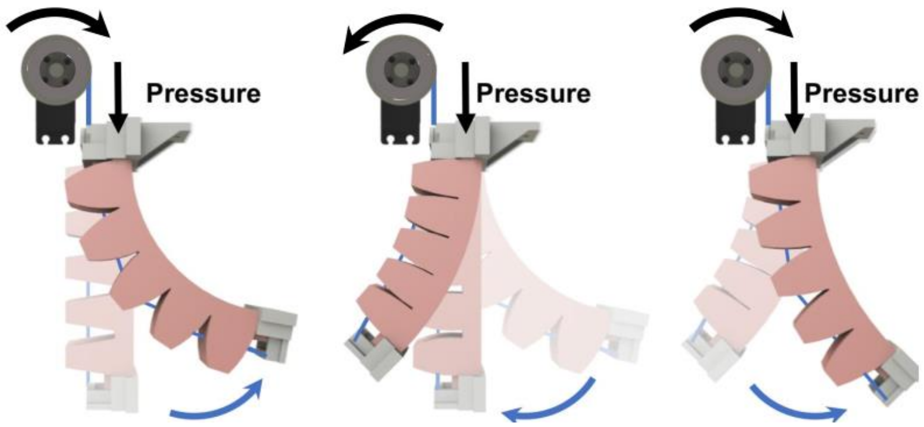

Figure S2: Illustration of the antagonistic actuation process, in which, the black markers represent the rotation of servo motors and the blue markers represent the motion of the actuator.

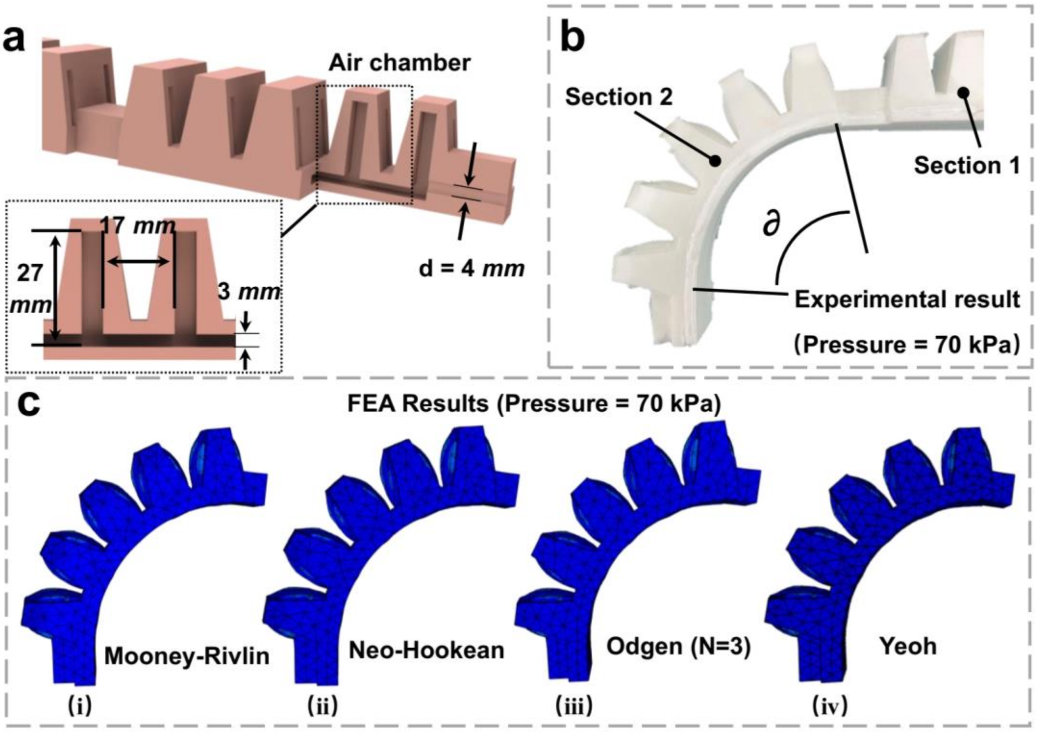

Figure S3: Finite element analysis of the soft actuator in penumatic mode. (a) Geometric parameters of the air chamber inside the antagonistic soft actuator. (b) Experimental bending state of the soft actuator at the inner pressure of 70 kPa. (c) Finite element analysis with 4 hyper-elastic model at the inner pressure of 70 kPa using ABAQUS.

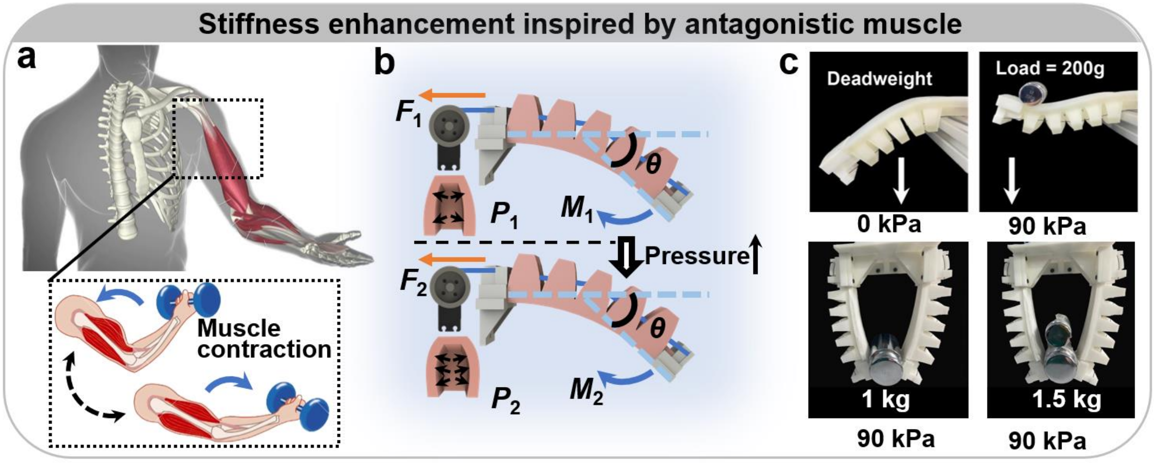

Figure S4: Stiffness enhancement inspired by antagonistic muscles contraction. (a) The schematic view of human muscle contraction. (b) Schematic and analysis of the stiffness enhancement. (c) Performance of stiffness enhancement.

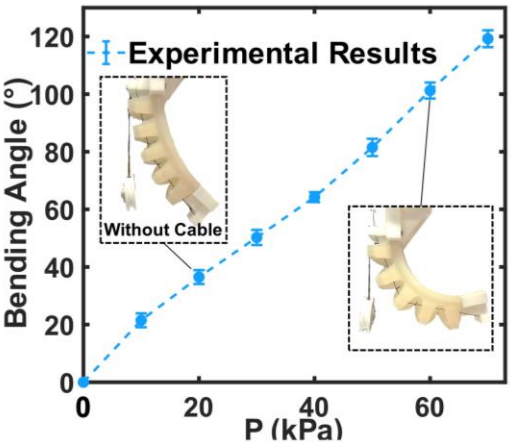

Figure S5: Measured bending angles of the soft bending actuator with penumatic actuaiton (without the cable for pulling) as a function of air pressure.

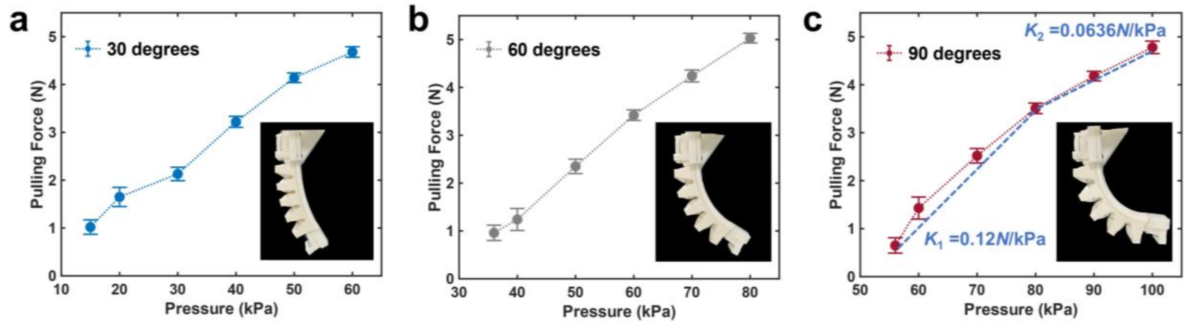

Figure S6: Measured cable pulling force of the antagonistic soft actuator as a function of pre-inflation pressusre at different fixed bending angles (30 degress, 60 degrees and 90 degrees from left to right).

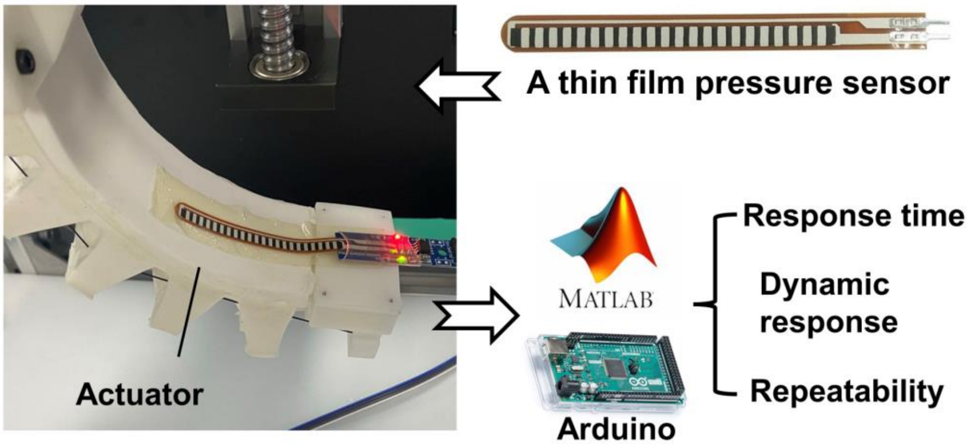

Figure S7: Schematic diagram of the actuator testing system.

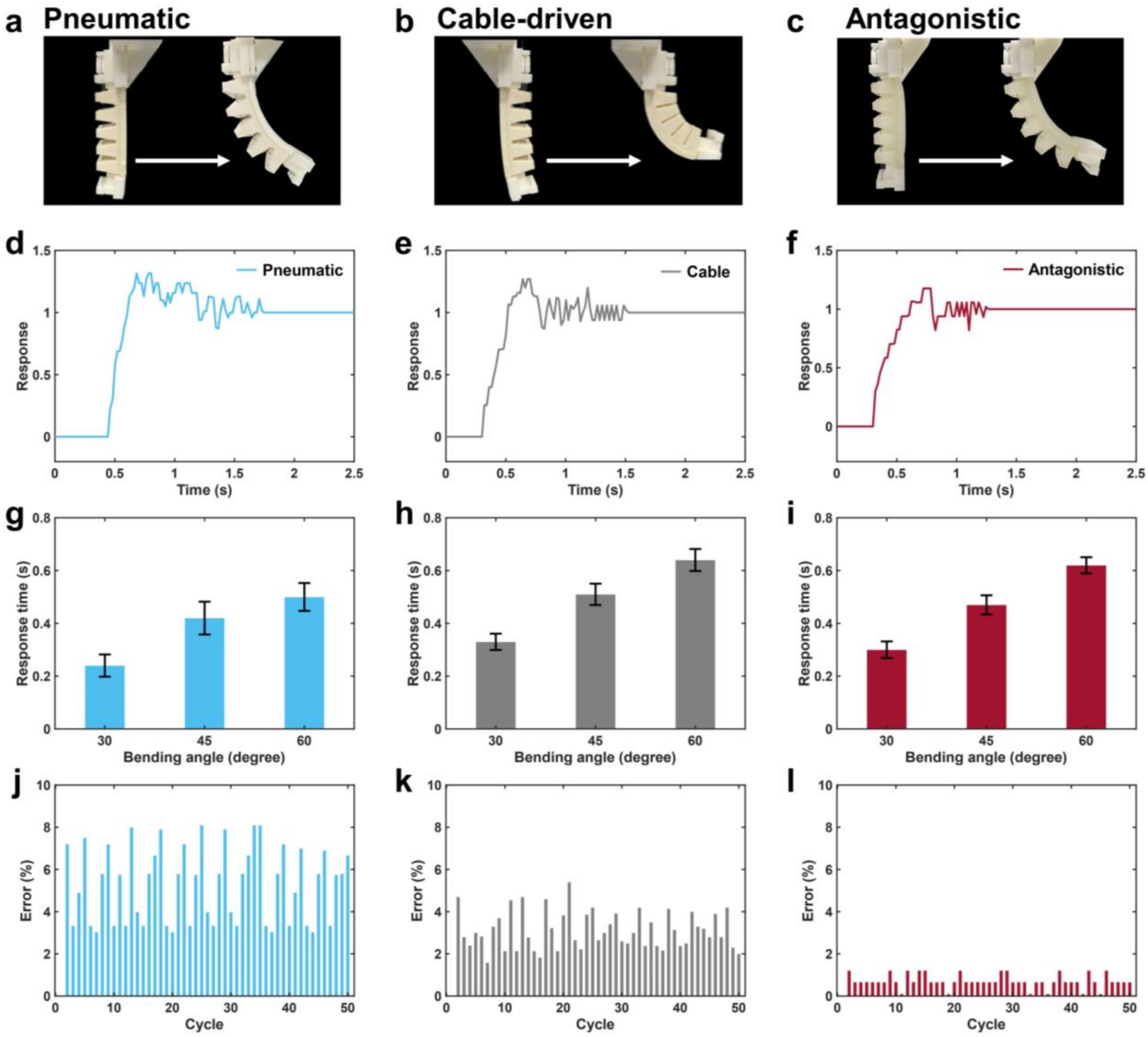

Figure S8: Chatacterization of the response performance for three actuation modes. (a) Soft pneumatic actuators. (b) Soft cable-driven actuators. (c) Soft antagonistic actuators. (d)-(f) measured dynamic response (normalized results) as a function of time for penumatic, cable-driven and antagonsitic actuators. (g) – (i) Measured response time as a function of bending angles (30 degrees, 45 degrees and 60 degrees) for penumatic, cable-driven and antagonsitic actuators. (j) – (l) Motionn repeatability tests in 50 cycles for penumatic, cable-driven and antagonsitic actuators.

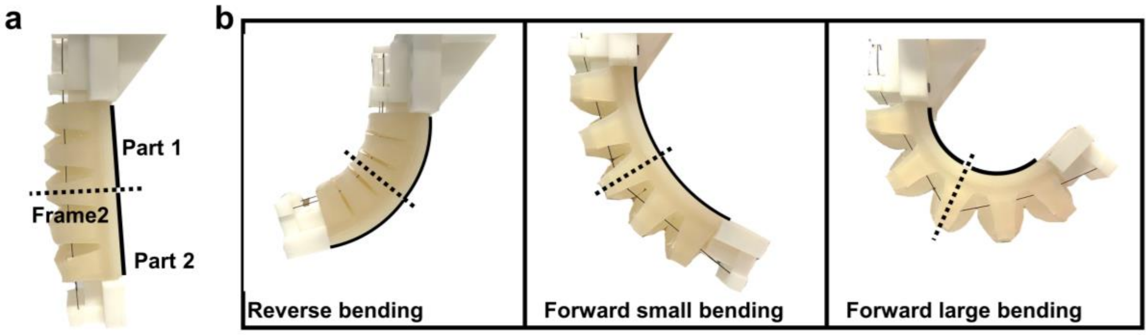

Figure S9: Bending validation of the antagonistic actuators. (a) The bending deformaiton of the actuator is divided into two parts by the black dotted line. (b) Actuatal bending behaviors of the actuator under different actuation states, including a reverse bending state, a forward small bending state and a forward large bending state.

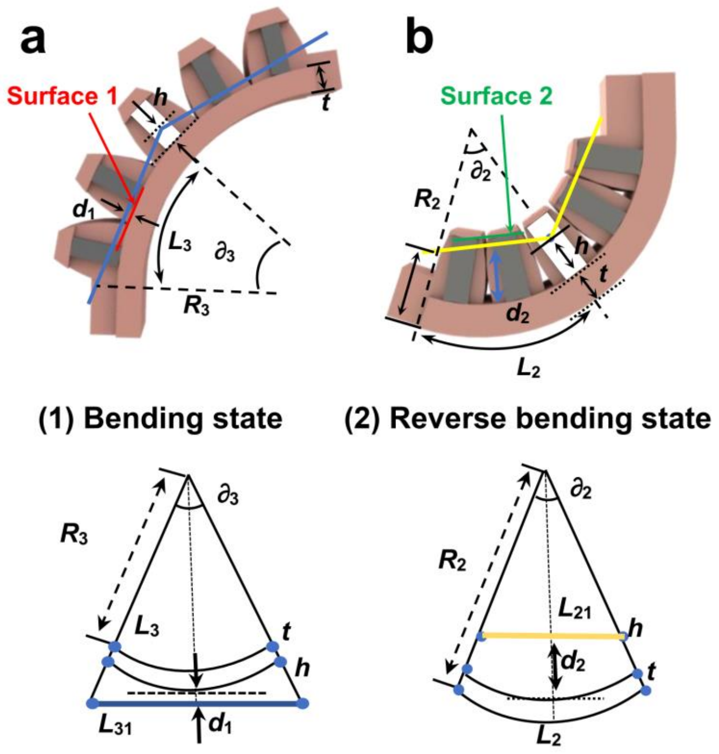

Figure S10: Schematic degisn of the Frame 2. (a) The bending state of the actuator. (b) The reverse bending state of the actuator.`

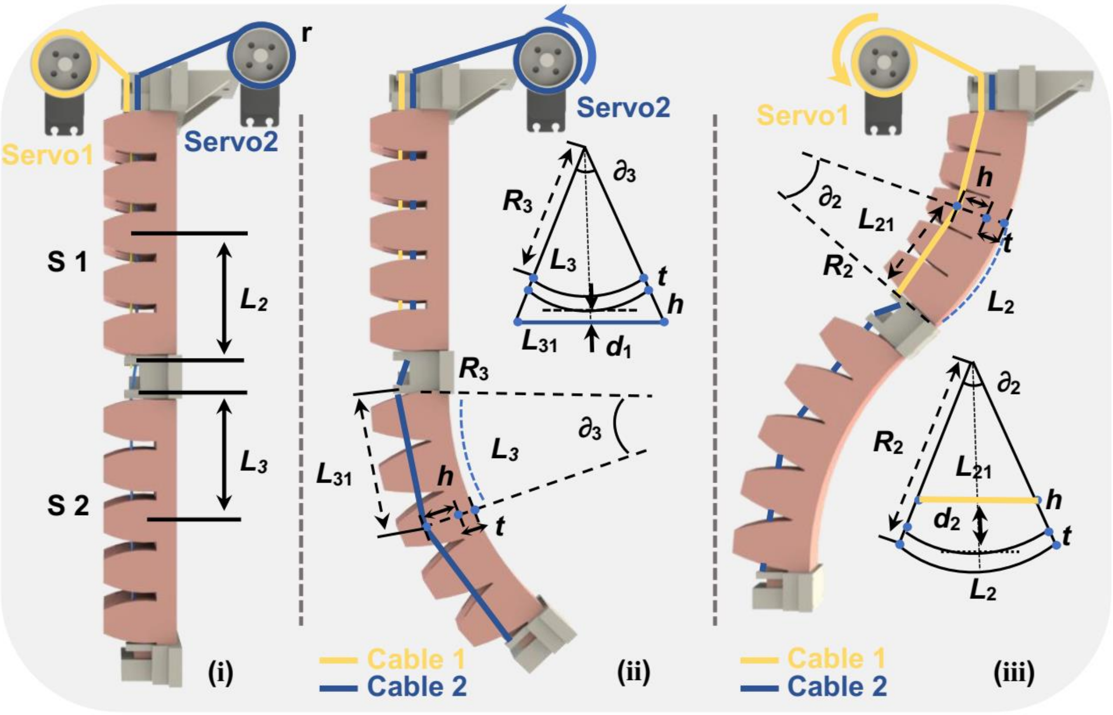

Figure S11: Kinematic modeling of the two-section soft continuum actuator. The relationship between the cable length variations and the actuator bending angles, (i) the neutral state; (ii) the (positive) bending state; (iii) the reverse bending state.

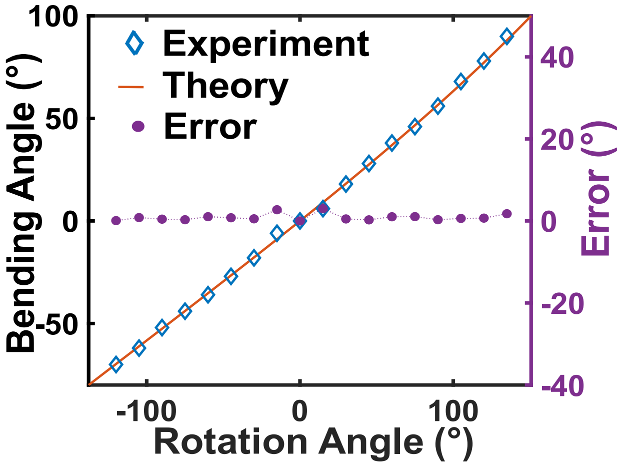

Figure S12: Theoretical and measured bending angles of the actuator as a function of servo rotation angles. The purple dots are the errors between the experiment and the theory.

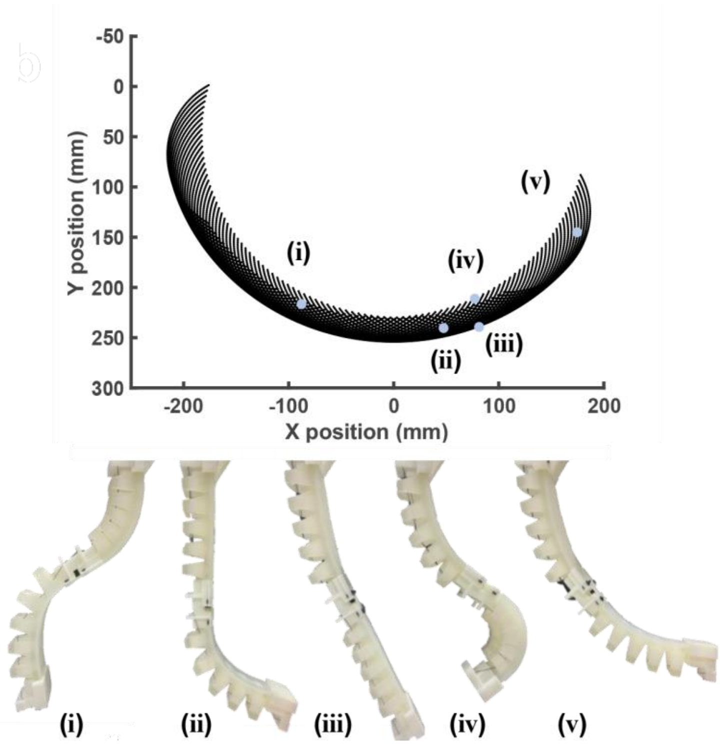

Figure S13: Working space analysis. The theoretical working space with the bending angle of section 1 is from -90 degrees to 50 degrees, and the bending angle of section 2 is from -90 degrees to 90 degrees. Several in-plane motion postures in the working space are shown as (i), (ii), (iii), (iv), and (v).

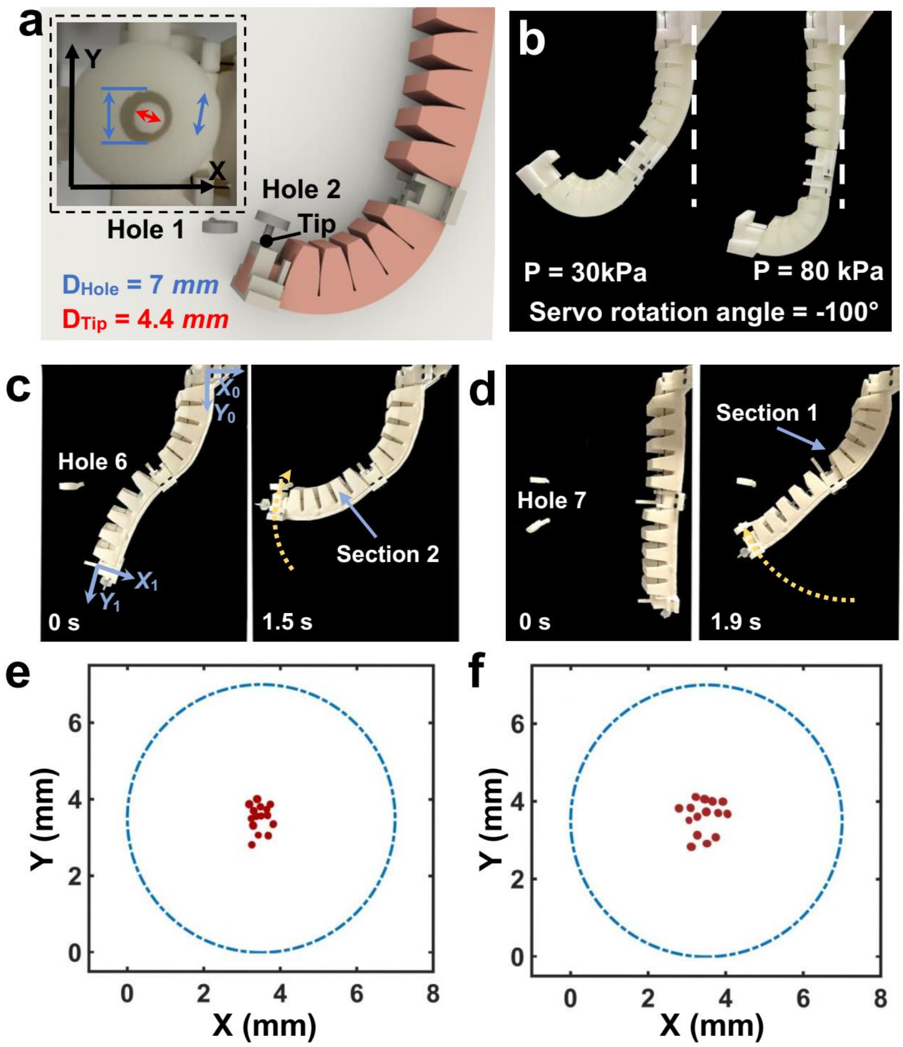

Figure S14: Stability verification after stiffness enhancement. (a) Schematic diagram of the soft robot for pin latching operation. (b) The improved stiffness greatly enhances the motion independence of the soft continuum actuator. (c) Pin latching operation (diameter 4.4 mm) for the Hole 6 (diameter 7 mm) by independently controlling the motion of the lower half section. (d) Pin latching operation for the Hole 7 by independently controlling the motion of the top half section. (e) Experimental results of the repetitive operation for Hole 6. (f) Experimental results of the repetitive operation for Hole 7.

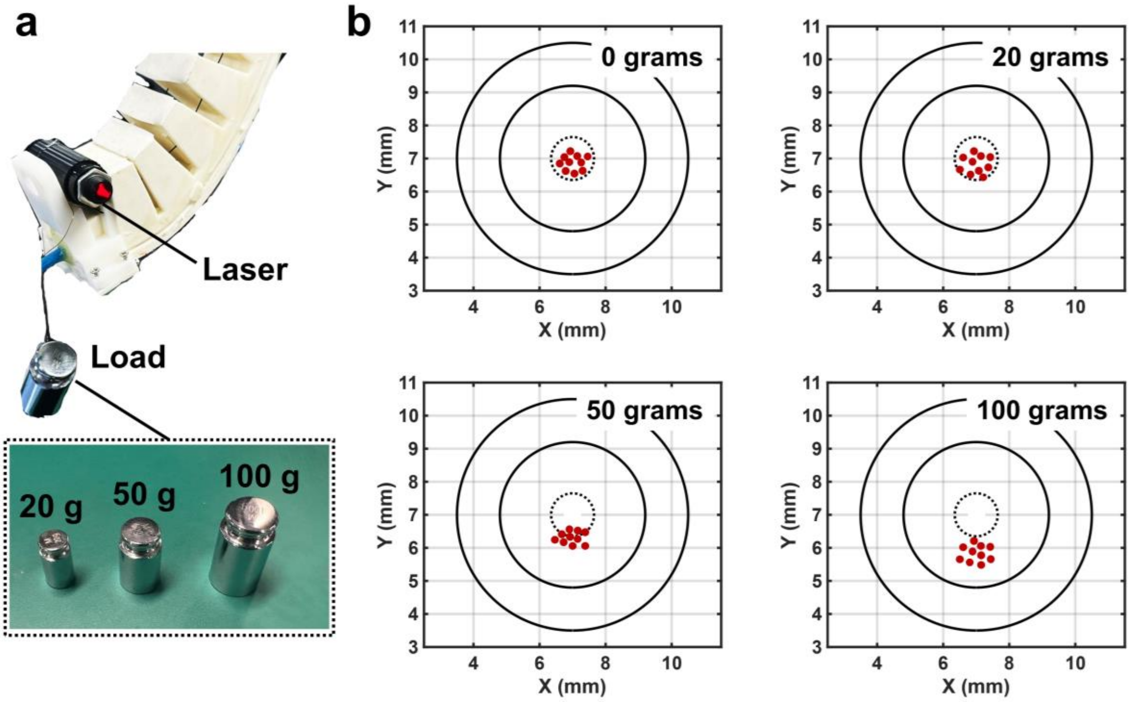

Figure S15: Motion accuracy testing under different end loads. (a) Repeatability tests using a red laser pointer. (b) Measured results of the Soft Co-bot in 10 cycles under different loads, including 0 grams, 20 grams, 50 grams and 100 grams.

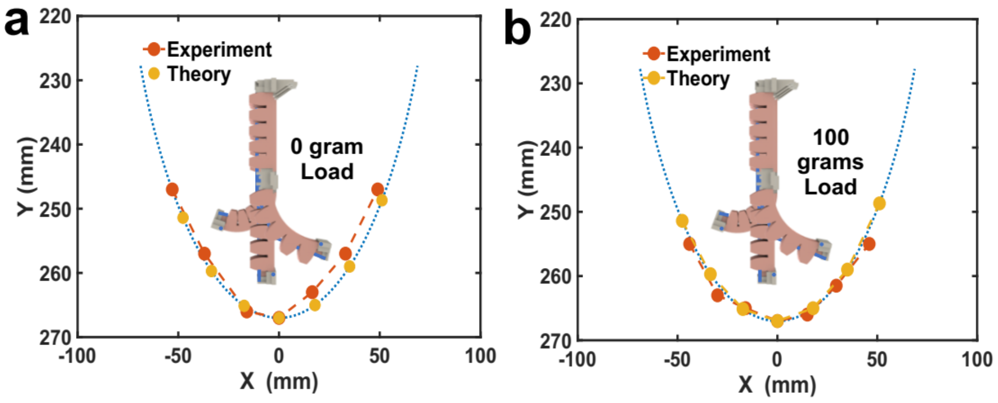

Figure S16: Point to point tracking test. (a) Experimental results with no external load based on the proposed kinematic model. (b) Experimental results with a 100-gram load at the end tip of the actuator.

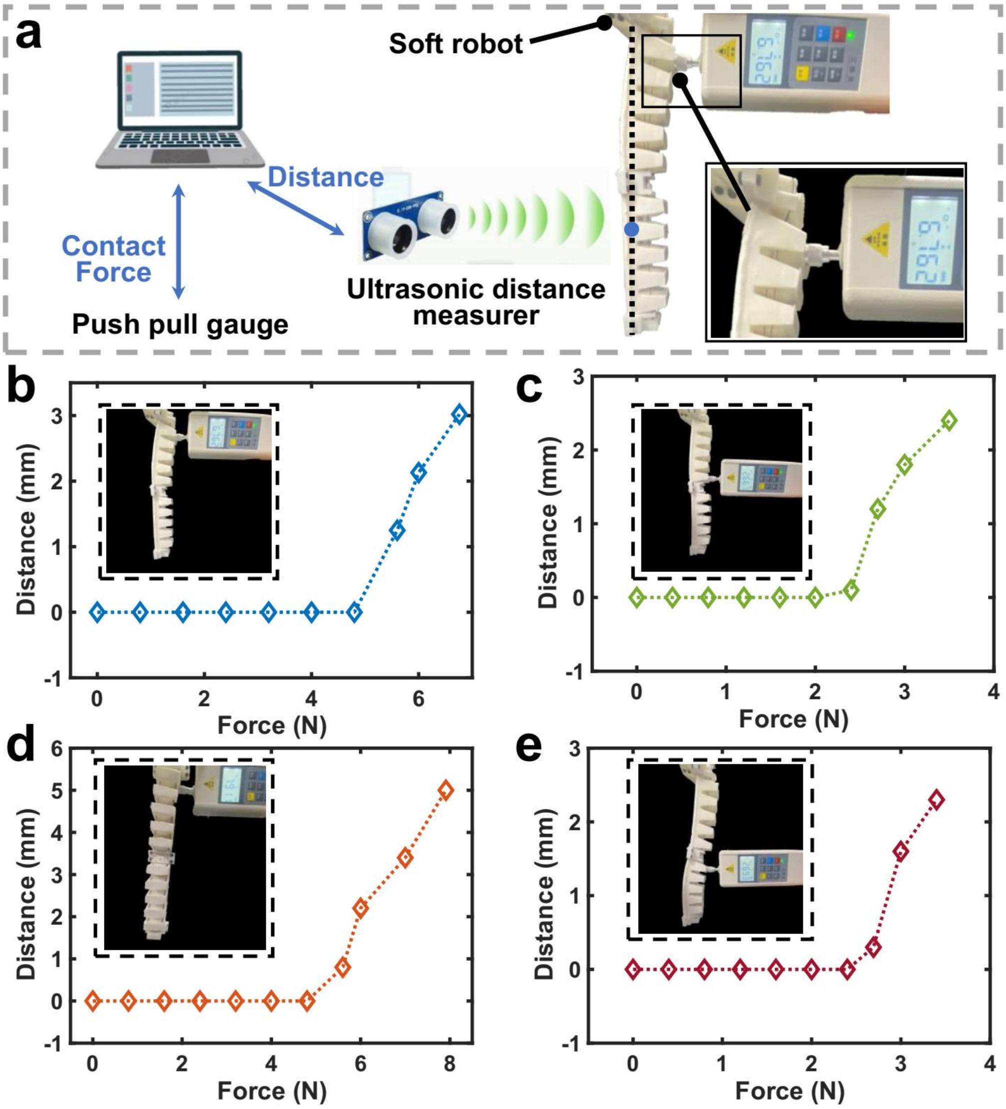

Figure S17: Stability characterization of the soft continuum actuator after stiffness enhancement via antagonistic actuation. (a) A schematic view of the test platform, consisting of a computer for data collection. A force gauge is used to provide the contact force and an ultrasonic distance sensor is used to record the passive movement of the actuator (marked point: the blue point). (b) – (e) Measured passive movement as a function of contact force for different measurement points.

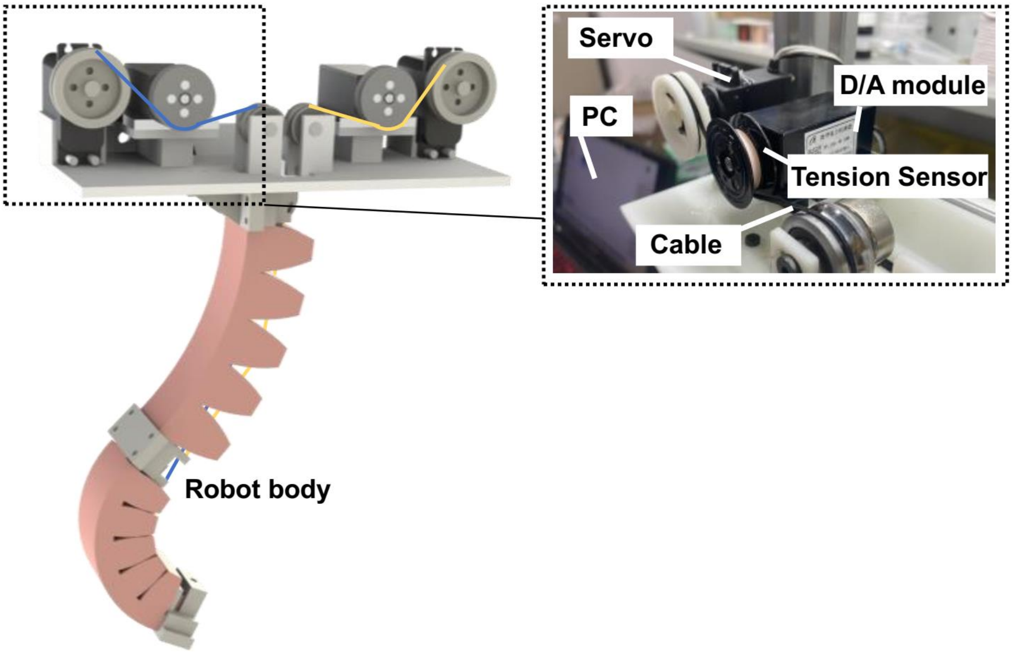

Figure S18: The prototype of the tension measurement system of the antagonistic soft continuum actuator for collaboration.

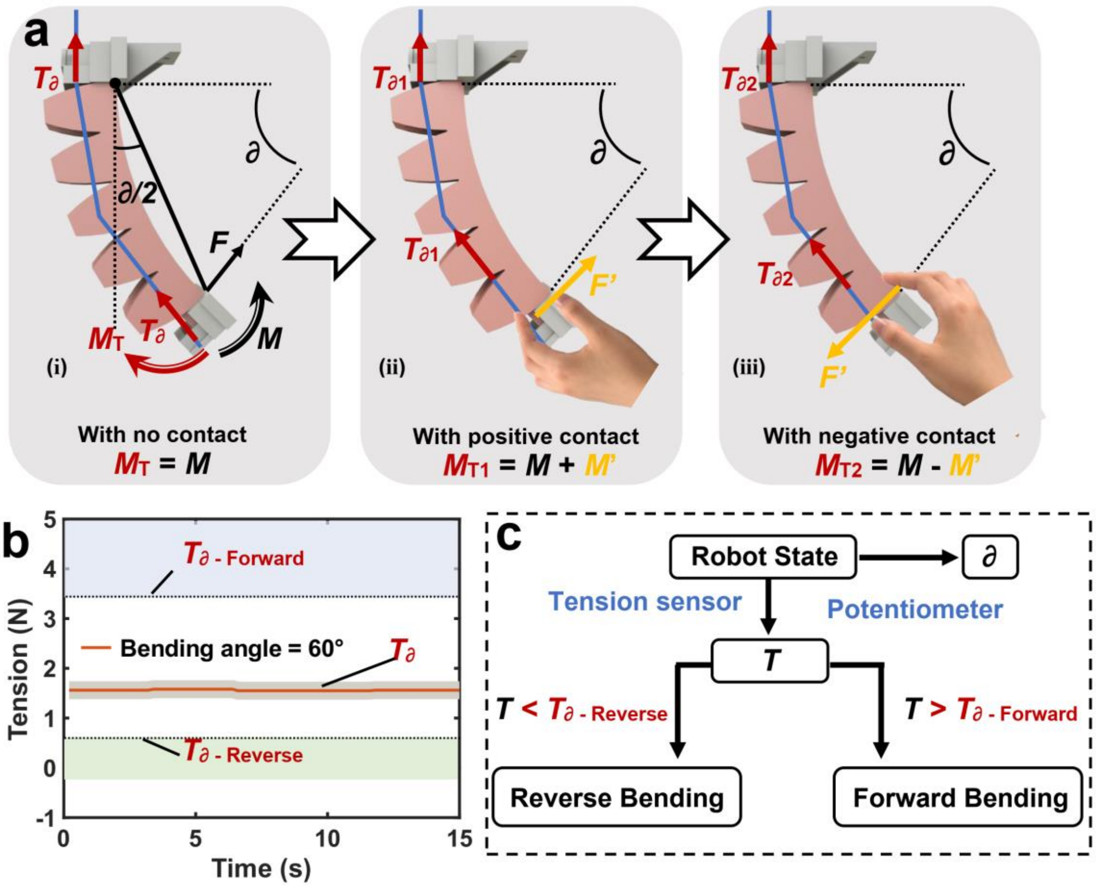

Figure S19: Schematic view for the judgement of the external dragging during collaborative operation. (a) Mechanical analysis of the actuator under different external drags. (b) Measured cable tension of the actuator at 60 degrees. The blue area represents the range for the driver to make a forward motion judgment at this bending angle with a critical value of $\boldsymbol{T}_{\partial\text{ - Forward}}$; the green range represents the range for the driver to make a reverse motion judgment at this bending angle with a critical value of $\boldsymbol{T}_{\partial\text{ - Reverse}}$. (c) Schematic diagram of the dragging direction decision.

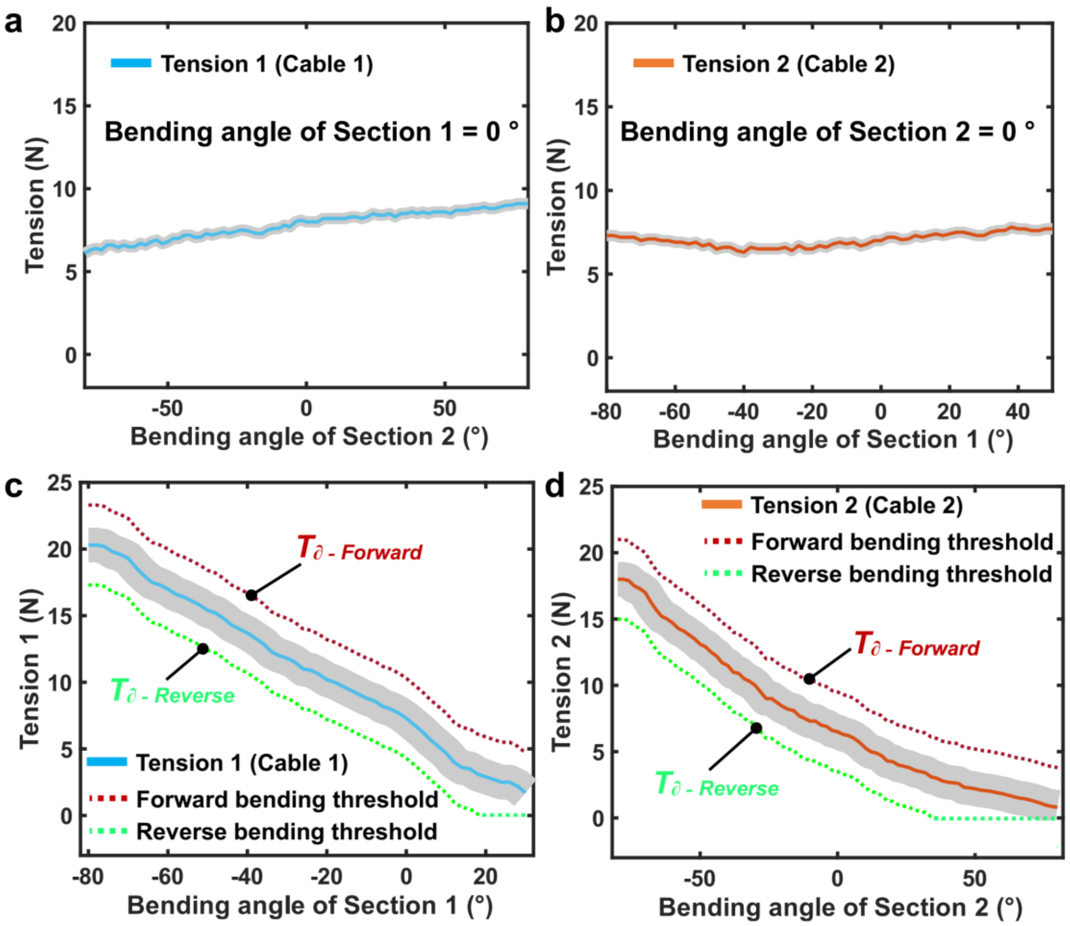

Figure S20: Cable tension measurement for the two-section soft continuum actuator. (a) Measured tension of cable 1 as a function of the bending angle of section 2 (the lower half section of the soft continuum actuator). When the bending angle of the section 1 (the upper half section of the soft continuum actuator) of the continuum actuator is 0 degree, and the gray area is the error bar. (b) Measured tension of cable 2 as a function of the bending angle of section 1, when the bending angle of the section 2 of the continuum actuator is 0 degree. (c) Measured tension of cable 1 as a function of the bending angle of the section 1 of the continuum actuator regardless of the section 2, where the red dotted curve represents the critical line for the section 1 to make the judgment of forward motion, and the green dotted curve represents the critical line for the section 1 to make the decision of reverse motion. (d) Measured tension of cable 2 as a function of the bending angle of the section 1 of the continuum actuator regardless of the section 1, where the red dotted line represents the critical curve for the section 2 to make the decision of forward motion, and the green dotted curve represents the critical threshold for the section 2 to make the decision of reverse motion.

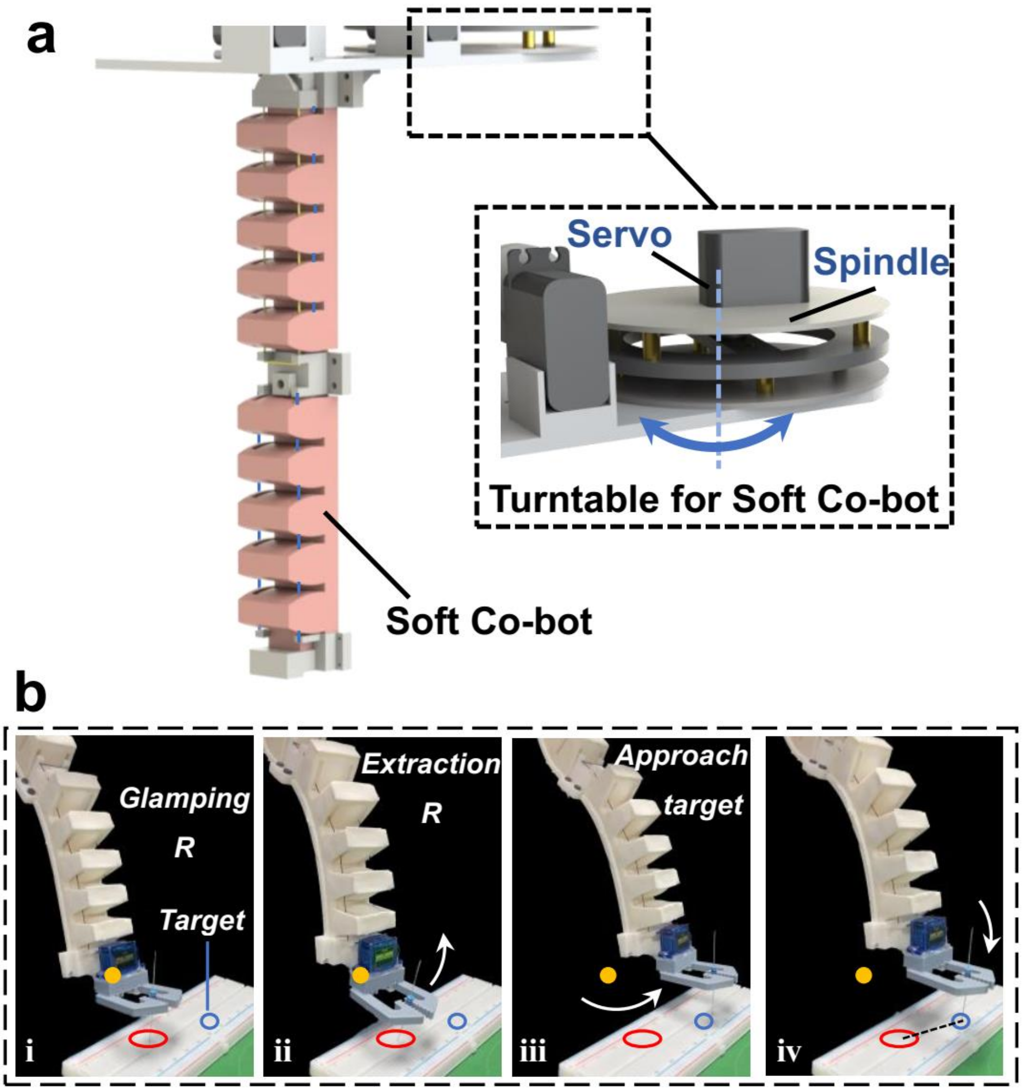

Figure S21: Turntable design for the Soft Co-bot. (a) Structure and installation of the turntable, to enlarge the robotic working space from 2D plane to 3D space. (b) The turntable assists the Soft Co-bot to transfer objects in 3D space.

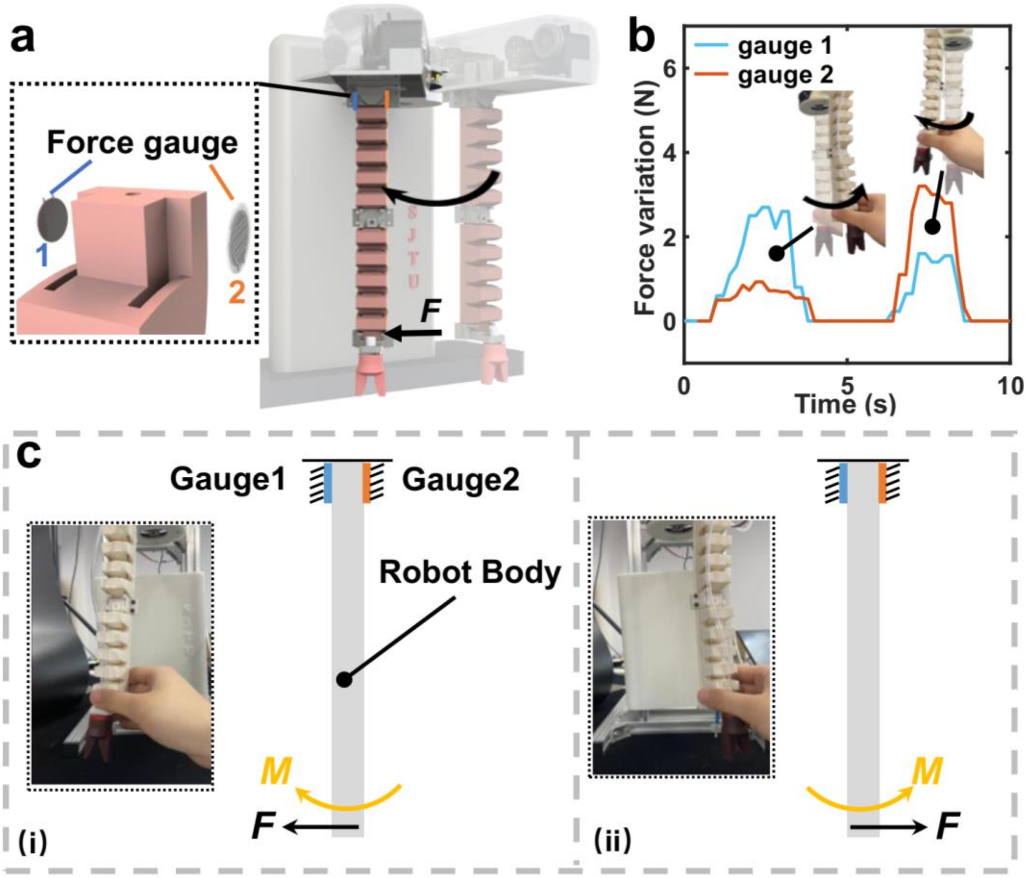

Figure S22: Principle of dragging collaboration for the turntable. (a) Two force gauges are mounted on the bottom of the Co-bot. (b) Measured force under different external dragging. (c) Schematic of the equivalent model when the end tip of the Co-bot is under dragging.

Supplementary Table S1

List of geometrical and materials parameters for the single soft actuator.

Parameter	Symbol	Value	Unit
Ecoflex-50 Nominal Young's modulus	E_{50}	35	kPa
Ecoflex-50 Density	ρ_{50}	1×10^{-9}	t/mm^3
Ecoflex-30 Nominal Young's modulus	E_{30}	21	kPa
Ecoflex-30 Density	ρ_{30}	1×10^{-9}	t/mm^3
Length of Soft actuator	L	106	mm
Cross-section dimension of the bottom layer	$w_b \times h_b$	32 × 9	mm
Cross-section dimension of the air chamber	$w_a \times h_a$	9 × 27	mm
Cross-section dimension of the narrow slot	$w_n \times h_n$	2 × 16.5	mm
Distance between bottom layer to chamber	d	3	mm

Supplementary Table S2

Constitutive model parameters (the units of parameters are expressed in MPa)

	**Ecoflex-30**	**Ecoflex-50**
**Mooney-Rivlin**	$C_{10} = 1.80\times10^{-2}$<br>$C_{01} = -4.69\times10^{-2}$<br>$C_{20} = 4.34\times10^{-5}$	$C_{10} = 3.53\times10^{-2}$<br>$C_{01} = -1.05\times10^{-1}$<br>$C_{20} = 4.77\times10^{-5}$
**Neo-Hookean**	$\mu = 0.03245$	$\mu = 0.048176$
**Odgen (N=3)**	$\mu_1 = -0322$, $\alpha_1 = 3.310$<br>$\mu_2 = 0.19$, $\alpha_2 = 3.115$<br>$\mu_3 = 0.145$, $\alpha_3 = 3.468$	$\mu_1 =1.970$, $\alpha_1 = 2.911$<br>$\mu_2 = -3.671$, $\alpha_2 = 3.008$<br>$\mu_3 = 1.740$, $\alpha_3 = 3.096$
**Yeoh**	$C_1 = 1.00\times10^{-1}$<br>$C_2 = 1.20\times10^{-2}$<br>$C_3 = 4.96\times10^{-5}$	$C_1 = 1.00\times10^{-1}$<br>$C_2 = 2.22\times10^{-2}$<br>$C_3 = 6.07\times10^{-5}$

Supplementary Table S3

Dynamic response performance comparison of different soft actuators

	**Response time**	**Steady-state error**	**Overshoot**	**Hysteresis**
This work	118 degrees/s	< 1.5 %	15%	< 2%
Cable-driven **[47]**	20 degrees/s	3.4%	18%	11%
Pneumatic **[11]**	254 degrees/s	8%	34%	16%
Antagonistic **[40]**	80 degrees/s	3.8%	21%	7%
SMA **[48]**	8 degrees/s	5%	8%	10%
DEA **[49]**	160 degrees/s	3%	13%	13%
